# Chemoselective *N*-acylation of indoles using thioesters as acyl source

**DOI:** 10.3762/bjoc.18.9

**Published:** 2022-01-10

**Authors:** Tianri Du, Xiangmu Wei, Honghong Xu, Xin Zhang, Ruiru Fang, Zheng Yuan, Zhi Liang, Yahui Li

**Affiliations:** 1 Key Laboratory of Agri-Food Safety of Anhui Province, School of Resources and Environment, Anhui Agricultural University, Hefei 230036, China

**Keywords:** indole, *N*-acylation, nucleophilic substitution, thioesters

## Abstract

The selective acylation of indoles often requires sensitive and reactive acyl chloride derivatives. Here, we report a mild, efficient, functional group tolerant, and highly chemoselective *N*-acylation of indoles using thioesters as a stable acyl source. A series of indoleamides have been obtained with moderate to good yields. In addition, heterocycles, such as carbazole, can also be used as nucleophiles in this reaction.

## Introduction

**Figure 1:**
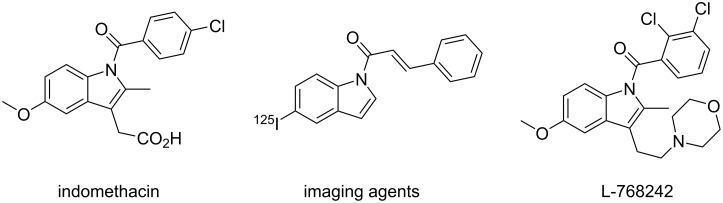
Representative pharmaceuticals containing *N*-acylindole moieties.

Molecules containing *N*-acylindoles have attracted wide attention in the synthetic polymers and pharmaceutical industry because of their unique structural, chemical, and biological properties [1]. For example, indomethacin is a nonselective inhibitor of COX1 and COX2, which is used for treating fever, pain and swelling [2]. Indole analog L-768242 exhibits nanomolar potencies (*K*_i_) with superior selectivity for the hCB2 receptor over the human central cannabinoid (hCB1) receptor [3] (Figure 1).

**Scheme 1:**
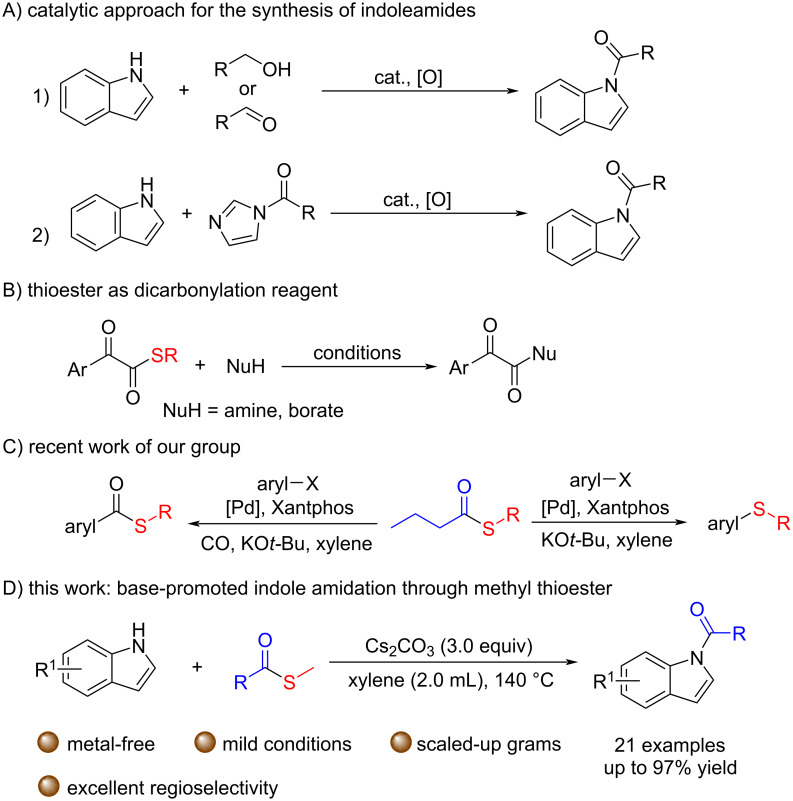
A) Strategies for the synthesis of *N*-acylindoles; B) thioester as dicarbonylation reagent; C) recent work of our group; D) this work.

Indole has multiple reactive sites, and chemoselective N- or C-functionalization of indoles is a challenging and important task [4–5]. Acylation of indoles frequently takes place at the C3 position because of the relatively strong electron cloud density. As *N*-acylated indoles are a widespread motif in many pharmaceuticals and natural products [6–8], selective *N*-acylation of indoles is very important. However, this process often requires unstable and reactive acyl chloride, which results in a poor functional group tolerance. Thus, developing a simple and efficient method for the synthesis of *N*-acylindoles becomes much attractive [9–12]. In 2009, Scheidt developed a dehydrogenative approach using indoles and alcohols catalyzed by tetrapropylammonium perruthenate [13] (Scheme 1, A1). In 2012, Sarpong successfully carried out chemoselective acylation of the N(sp^2^)–H bond by using a catalytic amount of DBU for the preparation of indoleamides [14] (Scheme 1, A2). Subsequently, Sundén reported an efficient chemoselective method for the synthesis of indoleamide by oxidative organocatalytic reaction of indole derivatives and conjugated aldehydes under NHC catalysis [15] (Scheme 1, A1).

In 2019, Jiang and co-workers reported a dicarbonylation of amine and aryl borates using α-ketothioester as a stable 1,2-dicarbonyl reagent [16] (Scheme 1, B). Recently, we applied *S*-methyl butanethioate in a Pd-catalyzed transthioetherification or transthioesterification of aryl halides for the synthesis of thioethers and thioesters [17] (Scheme 1, C). In addition, we also used this reagent to trap alkylcopper(I) intermediates and to form C−S bonds [18]. To the best of our knowledge, thioesters have not been developed as indole *N*-amidation reagent. Based on our continuous interest on thioesters, herein, we report an efficient and chemoselective protocol for the synthesis of indoleamide derivatives with thioesters as acyl source.

## Results and Discussion

At the beginning of our studies, we selected 3-methyl-1*H*-indole (**1a**) and *S*-methyl butanethioate (**2a**) as the model substrates to establish this procedure. As shown in Table 1, different bases were tried to improve the amidation reaction, and Cs_2_CO_3_ was found the most suitable choice (Table 1, entry 1). NaO*t*-Bu can also be used in this reaction and 82% yield could be obtained. In addition, NaOH and K_2_CO_3_ were not suitable in this procedure (Table 1, entries 3 and 4). Also the reaction did not work in the absence of Cs_2_CO_3_ (Table 1, entry 5). Solvent screening indicated that xylene was the best choice, and DMF, THF, and MeOH were not suitable for this reaction (Table 1, entries 7–9). When Cs_2_CO_3_ was reduced from 3.0 equiv to 2.0 equiv, 85% of the desired product could be obtained (Table 1, entry 10). Subsequently, we conducted a temperature optimization and 73% of the product was observed at 100 °C (Table 1, entry 11).

**Table 1:** Optimization of the reaction conditions^a^.

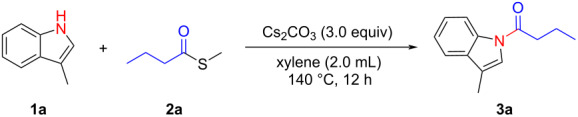		

Entry	Variation from standard conditions	Yield (%)^b^

1	none	97
2	NaO*t*-Bu instead of Cs_2_CO_3_	82
3	NaOH instead of Cs_2_CO_3_	trace
4	K_2_CO_3_ instead of Cs_2_CO_3_	trace
5	without Cs_2_CO_3_	NR^c^
6	toluene instead of xylene	89
7	DMF instead of xylene	0
8	THF instead of xylene	0
9	MeOH instead of xylene	0
10	2.0 equiv Cs_2_CO_3_	85
11	100 °C instead of 140 °C	73

^a^Reaction conditions: **1a** (0.2 mmol, 1.0 equiv), **2a** (0.6 mmol, 3.0 equiv), Cs_2_CO_3_ (0.6 mmol, 3.0 equiv), xylene (2.0 mL), 140 °C, 12 h. ^b^Yield was determined by GC using *n*-dodecane as the internal standard. ^c^NR = no reaction.

**Scheme 2:**
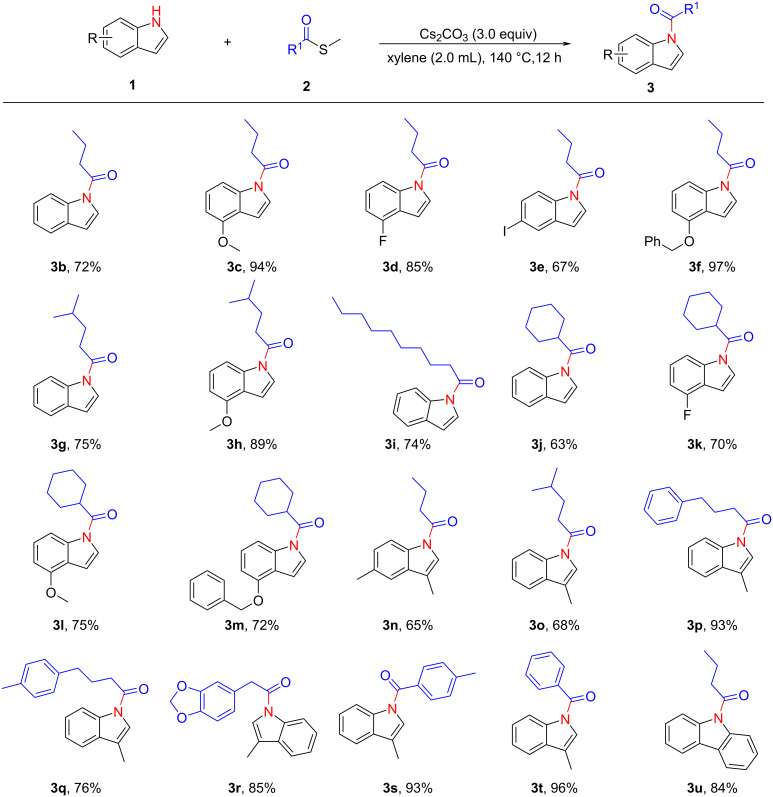
Reactions of thioesters and indoles. Reaction conditions: **1** (0.2 mmol, 1.0 equiv), **2** (0.6 mmol, 3.0 equiv), Cs_2_CO_3_ (0.6 mmol, 3.0 equiv), xylene (2.0 mL), 140 °C, 12 h. Isolated yields are shown.

**Scheme 3:**
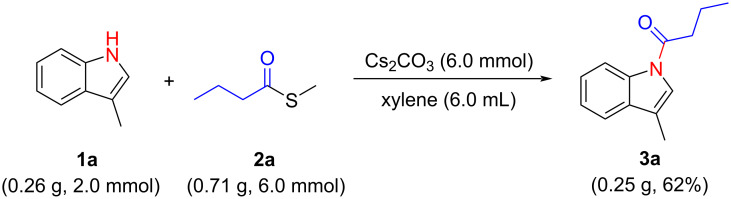
Gram-scale experiment.

Under the optimized reaction conditions, the scope of the reaction by variation of indoles and thioesters was tested. As shown in Scheme 2, a variety of functional groups, such as -OMe, -F and -I, are tolerated providing the desired products with moderate to excellent yields (Scheme 2, **3c**–**e**). In addition, various methyl thioesters could also participate in this reaction smoothly (Scheme 2, **3g**–**r**). Interestingly, *S*-methyl benzothioate and *S*-methyl 4-methylbenzothioate could also take part in this reaction and converted into the corresponding products **3s** and **3t** in 93% and 96% yield, respectively. Notably, carbazole could also be acylated with thioesters and 84% yield of the desired product was obtained (Scheme 2, **3u**).

With the established method for the *N*-acylation of indoles, a 2 mmol scale reaction was carried out. The reaction of 3-methyl-1*H*-indole (**1a**) and *S*-methyl butanethioate (**2a**) proceeded smoothly and 1-(3-methyl-1*H*-indol-1-yl)butan-1-one (**3a**) was obtained with 62% isolated yield (0.25 g, Scheme 3). The results indicate that this *N*-acylation reaction of indole has great potential in practical synthesis.

**Scheme 4:**
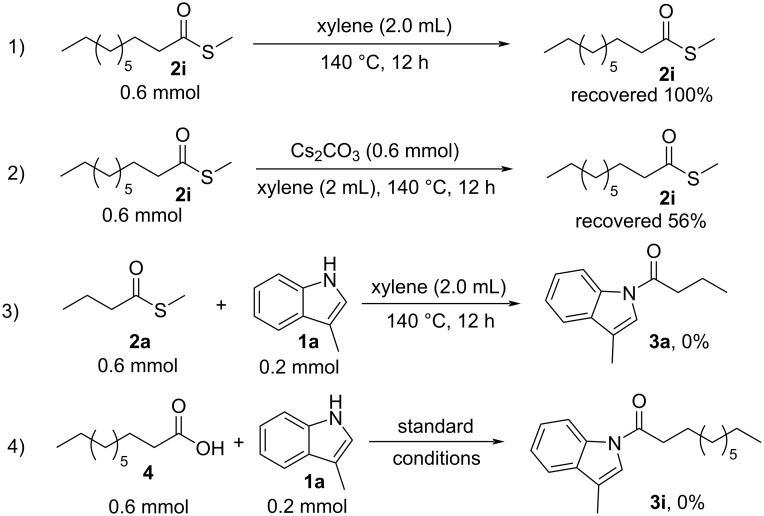
Control experiments.

Some control experiments were conducted to explore the reaction mechanism of this transformation (Scheme 4). When *S*-methyl decanethioate (**2i**) was adopted without Cs_2_CO_3_, no decomposition product was observed (Scheme 4, reaction 1). When *S*-methyl decanethioate (**2i**) was treated under the standard reaction conditions, 56% of **2i** was recovered (Scheme 4, reaction 2). Furthermore, without Cs_2_CO_3_, no desired product could be obtained (Scheme 4, reaction 3). These results indicate that Cs_2_CO_3_ plays an important role in the *N*-acylation process of indoles. The reaction of decanoic acid and 3-methyl-1*H*-indole (**1a**) was also conducted under the standard conditions, and no desired product was obtained, illustrating that 1-(3-methyl-1*H*-indol-1-yl)decan-1-one (**3i**) was not transformed from decanoic acid (**4**) (Scheme 4, reaction 4).

**Scheme 5:**
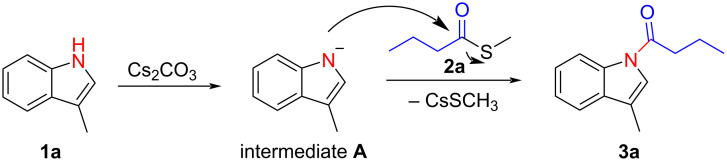
Proposed mechanism.

A plausible reaction mechanism has been proposed based on the results of the control experiments. As shown in Scheme 5, the reaction starts with a base-promoted deprotonation of indole forming intermediate **A**. In the next step nucleophilic substitution between intermediate **A** and **2a** occurs to give the desired *N*-acylindole product and CsSCH_3_ as byproduct [19–21] (Scheme 5).

## Conclusion

In conclusion, a chemoselective *N*-acylation of synthetically valuable indoles has been developed by using thioesters as a stable acyl source, a variety of *N*-acylated indoles could be obtained efficiently. Beside indole, carbazole can also take part in this reaction.

## Supporting Information

File 1 Experimental part and NMR spectra.
