# Supplementary material for: Chemoselective N-acylation of indoles using thioesters as acyl source
Source: Beilstein J Org Chem. 2022 Jan 10;18:89–94. doi: 10.3762/bjoc.18.9 (PMC8767559; doi:10.3762/bjoc.18.9)
Supplement: File 1 — Experimental part and NMR spectra. [file Beilstein_J_Org_Chem-18-89-s001.pdf]

BEILSTEIN JOURNAL OF ORGANIC CHEMISTRY

Supporting Information

for

Chemoselective *N*-acylation of indoles using thioesters as acyl source

Tianri Du, Xiangmu Wei, Honghong Xu, Xin Zhang, Ruiru Fang, Zheng Yuan, Zhi Liang and Yahui Li

Beilstein J. Org. Chem. **2022,** *18,* 89–94. doi:10.3762/bjoc.18.9

Experimental part and NMR spectra

License and Terms: This is a supporting information file under the terms of the Creative Commons Attribution License (https://creativecommons.org/licenses/by/4.0). Please note that the reuse, redistribution and reproduction in particular requires that the author(s) and source are credited and that individual graphics may be subject to special legal provisions.
The license is subject to the *Beilstein Journal of Organic Chemistry* terms and conditions: (https://www.beilstein-journals.org/bjoc/terms)

Contents

General information....S2

Control experiments....S2

General procedure for indoleamide....S3

Gram scale....S3

Product characterization....S4

References....S12

NMR spectra....S13

General information

Most of the chemicals were purchased from Aladdin, Energy-Chemical, TCI or Alfa Aesar and used as such unless stated otherwise. NMR spectra were recorded on Bruker Avance 600 and Bruker ARX 400 spectrometers. Chemical shifts (ppm) are given relative to solvent: references for chloroform-*d* were 7.26 ppm (^{1}H NMR) and 77.00 ppm (^{13}C NMR). Multiplets were assigned as s (singlet), d (doublet), t (triplet), q (quartet), p (pentet), dd (doublet of doublet), m (multiplet), and br. s (broad singlet). ^{19}F NMR spectra were recorded on a Varian 400 instrument spectrometer. Chemical shifts (δ) were given in parts per million (ppm) and were measured downfield from internal tetramethylsilane. GC-yields were calculated using isooctane as internal standard. All measurements were carried out at room temperature unless otherwise stated. GC-MS analysis was performed on a Shimadzu 2010 instrument and Rtx-5 capillary column. High resolution mass spectra (HRMS) were recorded on Agilent 6210. The data are given as mass units per charge (*m*/*z*). Gas chromatography analysis was performed on a Shimadzu 2010 instrument with a FID detector and Rtx-5 capillary column. The starting materials, *S*-methyl thiolates (except **2a**) were prepared according the previous literature.[1] The products were isolated from the reaction mixture by column chromatography on silica gel 60, 0.063–0.2 mm, 70–230 mesh (Merck).

Control experiments.

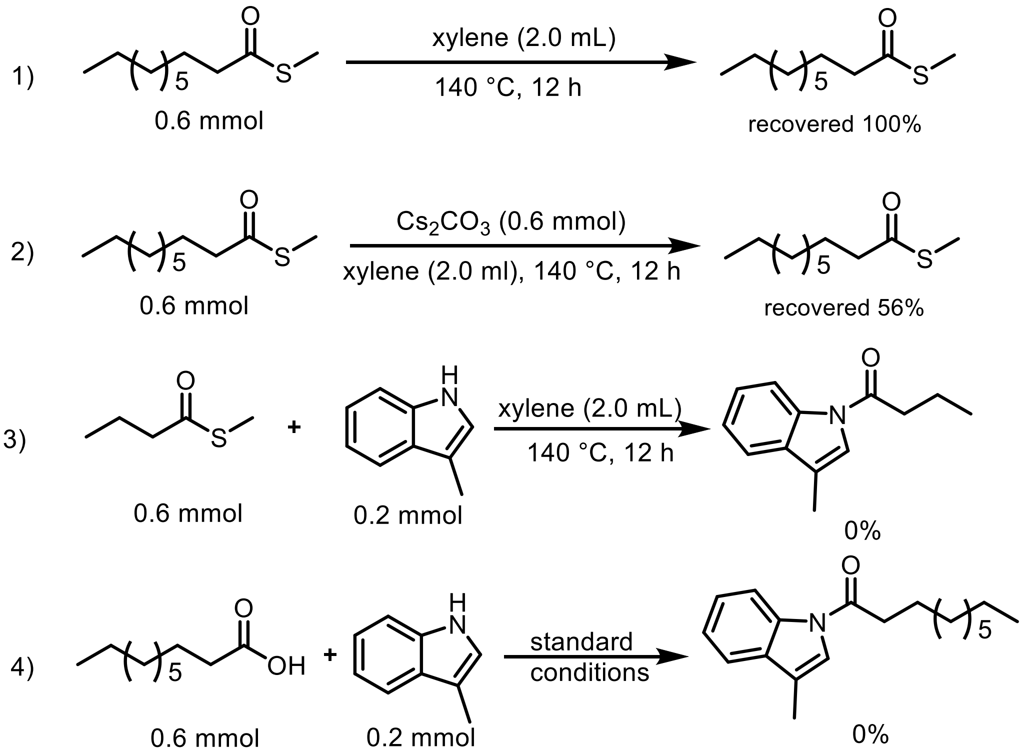

(1) A 25 mL tube was charged with *S*-methyl decanethioate (121.2 mg, 0.6 mmol) and xylene (2.0 mL) and an oven-dried stirring bar. The reaction was heated under 140 °C for 12 hours. Afterwards, the reaction was cooled to room temperature, and 50 μL *n*-

dodecane was added. Yield was determined by GC using *n*-dodecane as internal standard.

(2) A 25 mL tube was charged with *S*-methyl decanethioate (121.2 mg, 0.6 mmol, 1.0 equiv) and Cs_2CO_3 (195.6 mg, 0.6 mmol, 1.0 equiv) and xylene (2.0 mL) and an oven-dried stirring bar. The reaction was heated under 140 °C for 12 hours. Afterwards, the reaction is cooled to room temperature, and 50 μL *n*-dodecane was added. Yield was determined by GC using *n*-dodecane as internal standard.

(3) A 25 mL tube was charged with *S*-methyl butanethioate (70.8 mg, 0.6 mmol, 3.0 equiv), 3-methyl-1*H*-indole (26.2 mg, 0.2 mmol, 1.0 equiv), xylene (2.0 mL) and an oven-dried stirring bar. The reaction was heated under 140 °C for 12 hours. Afterwards, the reaction is cooled to room temperature, and 50 μL *n*-dodecane was added. Yield was determined by GC using *n*-dodecane as internal standard.

(4) A 25 mL tube was charged with decanoic acid (103.2 mg, 0.6 mmol, 3.0 equiv), 3-methyl-1*H*-indole (26.2 mg, 0.2 mmol, 1.0 equiv), xylene (2.0 mL) and an oven-dried stirring bar. The reaction was heated under 140 °C for 12 hours. Afterwards, the reaction is cooled to room temperature, and 50 μL *n*-dodecane was added. Yield was determined by GC using *n*-dodecane as internal standard.

General procedure for indoleamide

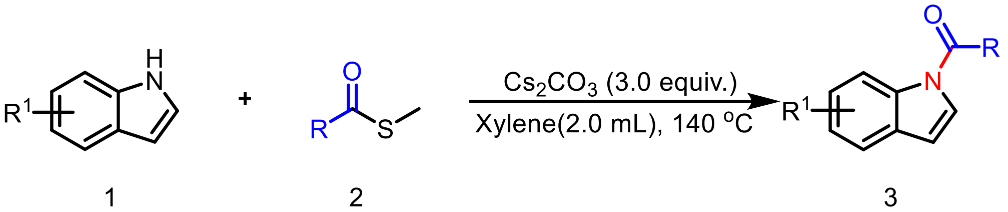

A 25 mL tube was charged with **1** (0.2 mmol, 1.0 equiv), **2** (0.6 mmol, 3.0 equiv), Cs_2CO_3 (0.6 mmol, 3.0 equiv) and xylene (2.0 mL) was added. The reaction was heated under 140 °C for 12 hours. Afterward, the reaction is cooled to room temperature. After removal of the solvent under reduced pressure, the pure product **3** was obtained by column chromatography on silica gel (eluent: pentane).

Gram scale

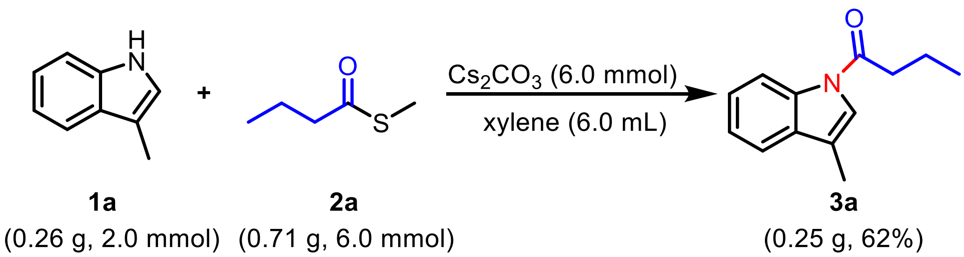

A 25 mL tube was charged with **1a** (0.26g, 2.0 mmol, 1.0 equiv), **2** (0.71g, 6.0

mmol, 1.0 equiv), Cs_2CO_3 (1.96 g, 6.0 mmol, 3.0 equiv) and xylene (6.0 mL) was added. The reaction was heated under 140 °C for 12 hours. Afterward, the reaction was cooled to room temperature. After removal of the solvent under reduced pressure, the pure product was obtained by column chromatography on silica gel (eluent: pentane).

Product Characterization

1-(3-Methyl-1*H*-indol-1-yl)butan-1-one (3a)[2]

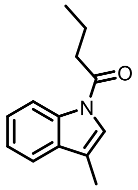

White solid, yield 97%

^{1}H NMR (600 MHz, Chloroform-*d*) δ = 8.48 – 8.40 (m, 1H), 7.50 (d, *J* = 7.6 Hz, 1H), 7.35 (t, *J* = 7.4 Hz, 1H), 7.29 (t, *J* = 7.2 Hz, 1H), 7.23 (s, 1H), 2.85 (t, *J* = 7.3 Hz, 2H), 2.29 (s, 3H), 1.87 (q, *J* = 7.4 Hz, 2H), 1.07 (t, *J* = 7.4 Hz, 3H).

^{13}C NMR (151 MHz, Chloroform-*d*) δ = 171.04, 135.91, 131.30, 125.07, 123.23, 121.59, 118.71, 118.11, 116.62, 37.75, 18.17, 13.79, 9.67.

1-(1*H*-indol-1-yl)butan-1-one (3b)[3]

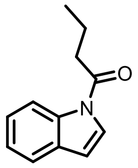

Colorless oil, yield 72%.

^{1}H NMR (600 MHz, Chloroform-*d*) δ = 8.48 (d, *J* = 8.1, 1H), 7.57 (d, *J* = 7.7, 1H), 7.47 (d, *J* = 3.3, 1H), 7.35 (t, *J* = 7.7, 1H), 7.30 – 7.24 (m, 1H), 6.64 (d, *J* = 3.6, 1H), 2.89 (t, *J* = 7.3, 2H), 1.88 (h, *J* = 7.3, 2H), 1.08 (t, *J* = 7.4, 3H).

^{13}C NMR (151 MHz, Chloroform-*d*) δ = 171.41, 135.61, 130.31, 124.99, 124.56, 123.48, 120.78, 116.62, 108.89, 37.73, 18.12, 13.74.

1-(4-Methoxy-1*H*-indol-1-yl)butan-1-one (3c)

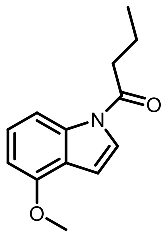

White solid, yield 94%

^{1}H NMR (600 MHz, Chloroform-*d*) δ = 8.07 (d, *J* = 8.3, 1H), 7.36 (d, *J* = 3.6, 1H), 7.29 – 7.26 (m, 1H), 6.76 (d, *J* = 3.7, 1H), 6.71 (d, *J* = 8.0, 1H), 3.94 (s, 3H), 2.87 (t, *J* = 7.3, 2H), 1.87 (h, *J* = 7.4, 2H), 1.07 (t, *J* = 7.4, 3H).

^{13}C NMR (151 MHz, Chloroform-*d*) δ = 171.55, 152.75, 136.84, 125.95, 123.03, 120.53, 109.69, 105.89, 103.98, 55.34, 37.76, 18.08, 13.72.
HRMS (ESI) calcd for $[M + H]^+$ ($C_{13}H_{16}O_2N$), 218.1176; found, 218.1175.

1-(4-Fluoro-1*H*-indol-1-yl)butan-1-one (3d)

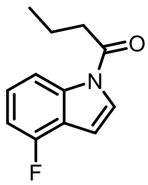

White solid, yield 85%
^{1}H NMR (600 MHz, Chloroform-*d*) δ = 8.24 (d, *J* = 8.3, 1H), 7.44 (d, *J* = 3.8, 1H), 7.31 – 7.26 (m, 1H), 6.98 – 6.92 (m, 1H), 6.74 (d, *J* = 3.8, 1H), 2.89 (t, *J* = 7.3, 2H), 1.91 – 1.84 (m, 2H), 1.07 (t, *J* = 7.4, 3H).
^{13}C NMR (151 MHz, Chloroform-*d*) δ = 171.38, 155.60 (*J* = 124.58), 125.87, 125.82, 124.50, 112.66, 108.87, 108.75, 104.39, 37.74, 18.02, 13.67.
^{19}F NMR (564 MHz, Chloroform-*d*) δ = -122.11.
HRMS (ESI) calcd for $[M + H]^+$ ($C_{12}H_{13}ONF$), 206.0976; found, 206.0975.

1-(5-Iodo-1*H*-indol-1-yl)butan-1-one (3e)

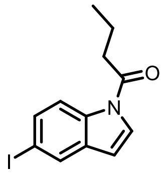

White solid, yield 67%
^{1}H NMR (600 MHz, Chloroform-*d*) δ = 8.23 (d, *J* = 8.7, 1H), 7.89 (d, *J* = 1.0, 1H), 7.61 – 7.60 (m, 1H), 7.41 (d, *J* = 3.5, 1H), 6.54 (d, *J* = 3.5, 1H), 2.86 (t, *J* = 7.3, 2H), 1.92 – 1.80 (m, 2H), 1.07 (t, *J* = 7.4, 3H).
^{13}C NMR (151 MHz, Chloroform-*d*) δ = 171.30, 134.85, 133.48, 132.56, 129.60, 125.29, 118.41, 107.84, 87.63, 37.61, 18.01, 13.74.
HRMS (ESI) calcd for $[M + H]^+$ ($C_{12}H_{13}ONI$), 314.0036; found, 314.0033.

1-(4-(Benzyloxy)-1*H*-indol-1-yl)butan-1-one (3f)

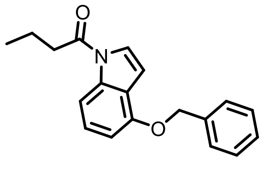

White solid, yield 97%
^{1}H NMR (600 MHz, Chloroform-*d*) δ 8.11 (d, *J* = 8.2 Hz, 1H), 7.50 (d, *J* = 7.5 Hz, 2H), 7.42 (t, *J* = 7.5 Hz, 2H), 7.39 – 7.33 (m, 2H), 7.27 (t, *J* = 8.2 Hz, 1H), 6.84 (d, *J* = 3.6 Hz, 1H), 6.79 (d, *J* = 8.0 Hz, 1H), 5.21 (s, 2H), 2.88 (t, *J* = 7.3 Hz, 2H), 1.88 (h, *J* = 7.4 Hz, 2H), 1.09 (t, *J* = 7.4 Hz, 3H).

^{13}C NMR (151 MHz, Chloroform-*d*) δ = 171.57, 151.92, 137.16, 136.95, 128.54, 127.90, 127.36, 125.96, 123.14, 120.96, 109.99, 106.12, 105.53, 70.11, 37.77, 18.09, 13.77.
HRMS (ESI) calcd for [M + H]$^+$ ($C_{19}H_{20}O_2N$), 294.1489; found, 294.1487.

1-(1*H*-indol-1-yl)-4-methylpentan-1-one (3g)

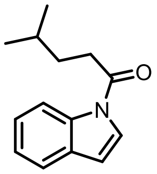

Yellow solid, yield 75%
^{1}H NMR (600 MHz, Chloroform-*d*) δ = 8.46 (d, *J* = 8.3, 1H), 7.56 (d, *J* = 7.7, 1H), 7.47 (d, *J* = 3.7, 1H), 7.37 – 7.32 (m, 1H), 7.28 – 7.26 (m, 1H), 6.63 (d, *J* = 3.7, 1H), 2.94 – 2.86 (m, 2H), 1.78 – 1.67 (m, 3H), 0.98 (d, *J* = 6.4, 6H).
^{13}C NMR (151 MHz, Chloroform-*d*) δ = 171.73, 135.64, 130.31, 125.03, 124.59, 123.49, 120.72, 116.60, 108.91, 33.90, 33.51, 27.68, 22.30.
HRMS (ESI) calcd for [M + Na]$^+$ ($C_{14}H_{17}ONNa$), 238.1202; found, 238.1200.

1-(4-Methoxy-1*H*-indol-1-yl)-4-methylpentan-1-one (3h)

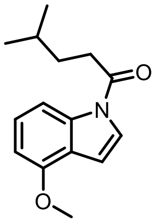

White solid, yield 89%
^{1}H NMR (600 MHz, Chloroform-*d*) δ = 8.05 (d, *J*=8.3, 1H), 7.37 (d, *J*=3.7, 1H), 7.26 (d, *J* = 10.1, 1H), 6.76 (d, *J* = 3.7, 1H), 6.71 (d, *J*=8.0, 1H), 3.93 (s, 3H), 2.93 – 2.85 (m, 2H), 1.77 – 1.66 (m, 3H), 0.97 (d, *J* = 6.3, 6H).
^{13}C NMR (151 MHz, Chloroform-*d*) δ = 171.87, 152.75, 136.87, 125.98, 123.05, 120.55, 109.70, 105.92, 104.00, 55.39, 33.94, 33.47, 27.67, 22.28.
HRMS (ESI) calcd for [M + Na]$^+$ ($C_{15}H_{19}O_2NNa$), 268.1308; found, 268.1306.

1-(1*H*-Indol-1-yl)decan-1-one (3i)

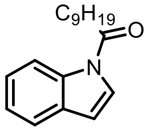

Colorless oil, yield 74%.
^{1}H NMR (600 MHz, Chloroform-*d*) δ = 8.47 (d, *J* = 8.2 Hz, 1H), 7.56 (d, *J* = 7.7 Hz, 1H), 7.45 (d, *J* = 3.6 Hz, 1H), 7.35 (t, *J* = 7.7 Hz, 1H), 7.32 – 7.22 (m, 1H), 6.62 (d, *J* = 3.7 Hz, 1H), 2.89 (t, *J* = 7.5 Hz, 2H), 1.86 – 1.81 (m, 2H), 1.46 – 1.41 (m, 2H), 1.39 – 1.34 (m, 2H), 1.37 – 1.23 (m, 8H), 0.89 (t, *J* = 6.9 Hz, 3H).

^{13}C NMR (151 MHz, Chloroform-*d*) δ = 171.55, 135.64, 130.33, 125.00, 124.61, 123.48, 120.71, 116.60, 108.87, 35.88, 31.82, 29.39, 29.35, 29.21, 24.69, 22.61, 14.02.

Cyclohexyl(1*H*-indol-1-yl)methanone (3j)[4]

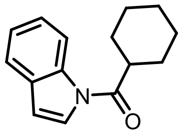

Pink solid, yield 63%
^{1}H NMR (600 MHz, Chloroform-*d*) δ 8.49 (d, *J* = 8.3 Hz, 1H), 7.55 (d, *J* = 7.7 Hz, 1H), 7.50 (d, *J* = 3.7 Hz, 1H), 7.34 (t, *J* = 7.8 Hz, 1H), 7.28 – 7.24 (m, 1H), 6.63 (d, *J* = 3.5 Hz, 1H), 3.00 (tt, *J* = 11.5, 3.3 Hz, 1H), 2.01 (d, *J* = 12.5 Hz, 2H), 1.93 – 1.85 (m, 2H), 1.80 – 1.64 (m, 3H), 1.48 – 1.29 (m, 3H).
^{13}C NMR (151 MHz, Chloroform-*d*) δ = 174.82, 135.71, 130.38, 125.02, 124.52, 123.48, 120.62, 116.78, 108.81, 43.74, 29.56, 25.72, 25.62.

Cyclohexyl(4-fluoro-1*H*-indol-1-yl)methanone (3k)

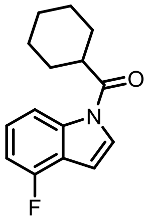

White solid, yield 70%
H NMR (600 MHz, Chloroform-*d*) δ 8.26 (d, *J* = 8.3 Hz, 1H), 7.47 (d, *J* = 3.7 Hz, 1H), 7.29 – 7.24 (m, 1H), 6.97 – 6.92 (m, 1H), 6.74 (d, *J* = 3.5 Hz, 1H), 2.99 (tt, *J* = 11.5, 3.2 Hz, 1H), 2.01 (d, *J* = 12.6 Hz, 2H), 1.89 (d, *J* = 13.3 Hz, 2H), 1.77 (d, *J* = 12.7 Hz, 1H), 1.69 (qd, *J* = 13.2, 2.9 Hz, 2H), 1.45 – 1.36 (m, 2H), 1.35 – 1.30 (m, 1H).
^{13}C NMR (151 MHz, Chloroform-*d*) δ = 174.84, 155.55 (*J* = 124.58), 137.74, 125.84, 124.46, 119.20, 112.83, 108.84, 104.30, 43.81, 29.51, 25.66, 25.56.
HRMS (ESI) calcd for $[M + H]^+$ ($C_{15}H_{17}ONF$), 246.1289; found, 246.1287.

Cyclohexyl(4-methoxy-1*H*-indol-1-yl)methanone (3l)

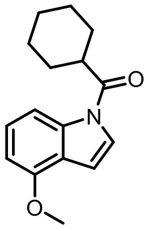

White solid, yield 75%
^{1}H NMR (600 MHz, Chloroform-*d*) δ 8.08 (d, *J* = 8.3 Hz, 1H), 7.41 (d, *J* = 3.7 Hz, 1H), 7.27 (t, *J* = 8.2 Hz, 1H), 6.77 (d, *J* = 3.7 Hz, 1H), 6.71 (d, *J* = 8.0 Hz, 1H), 3.94 (s, 3H), 3.00 (tt, *J* = 11.6, 3.2 Hz, 1H), 2.00 (d, *J* = 12.5 Hz, 2H), 1.88 (d, *J* = 13.3 Hz, 2H), 1.76

(d, *J* = 12.6 Hz, 1H), 1.68 (qd, *J* = 13.1, 2.9 Hz, 2H), 1.44 – 1.35 (m, 2H), 1.35 – 1.29 (m, 1H).
^{13}C NMR (151 MHz, Chloroform-*d*) δ = 174.96, 152.68, 136.93, 125.96, 122.99, 120.61, 109.88, 105.81, 103.98, 55.40, 43.80, 29.53, 25.72, 25.62.
HRMS (ESI) calcd for [M + Na$^+$]$^+$ ($C_{16}H_{19}O_2NNa$), 280.1308; found, 280.1308.

(4-(Benzyloxy)-1*H*-indol-1-yl)(cyclohexyl)methanone (3m)

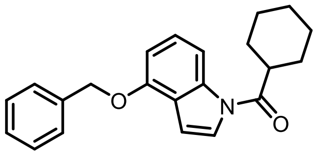

White solid, yield 72%
^{1}H NMR (600 MHz, Chloroform-*d*) δ = 8.10 (d, *J* = 8.3 Hz, 1H), 7.48 (d, *J* = 7.4 Hz, 2H), 7.43 – 7.38 (m, 3H), 7.33 (t, *J* = 7.3 Hz, 1H), 7.28 – 7.22 (m, 1H), 6.83 (d, *J* = 3.7 Hz, 1H), 6.77 (d, *J* = 8.0 Hz, 1H), 5.21 (s, 2H), 3.02 – 2.99 (m, 1H), 2.01 (d, *J* = 12.4 Hz, 2H), 1.91 – 1.88 (m, 2H), 1.77 (d, *J* = 12.5 Hz, 1H), 1.73 – 1.66 (m, 2H), 1.47 – 1.26 (m, 3H).
^{13}C NMR (151 MHz, Chloroform-*d*) δ = 174.96, 151.85, 137.18, 137.05, 128.50, 127.85, 127.31, 125.93, 123.06, 121.04, 110.17, 106.03, 105.57, 70.16, 43.79, 29.55, 25.73, 25.62.
HRMS (ESI) calcd for [M + H]$^+$ ($C_{22}H_{24}O_2N$), 334.1802; found, 334.1801.

1-(3,5-Dimethyl-1*H*-indol-1-yl)butan-1-one (3n)

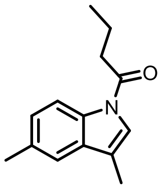

White solid, yield 65%
^{1}H NMR (600 MHz, Chloroform-*d*) δ = 8.31 (d, *J* = 5.8, 1H), 7.27 (d, *J* = 13.1, 1H), 7.20 – 7.14 (m, 2H), 2.82 (t, *J* = 7.4, 2H), 2.46 (s, 3H), 2.26 (d, *J* = 1.1, 3H), 1.86 (h, J = 7.4 Hz, 2H), 1.06 (t, *J* = 7.4, 3H).
^{13}C NMR (151 MHz, Chloroform-*d*) δ = 170.80, 134.12, 132.75, 131.53, 126.27, 121.69, 118.68, 117.86, 116.25, 37.64, 21.34, 18.19, 13.76, 9.60.

4-Methyl-1-(3-methyl-1*H*-indol-1-yl)pentan-1-one (3o)

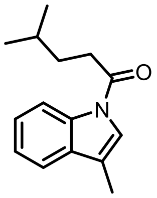

Colorless oil, yield 68%

^{1}H NMR (600 MHz, Chloroform-*d*) δ = 8.44 (d, *J* = 7.7, 1H), 7.49 (d, *J* = 7.6, 1H), 7.36 – 7.33 (m, 1H), 7.30 – 7.24 (m, 1H), 7.22 (s, 1H), 2.89 – 2.81 (m, 2H), 2.28 (d, *J* = 1.2, 3H), 1.77 – 1.66 (m, 3H), 0.97 (t, *J* = 6.6, 6H).
^{13}C NMR (151 MHz, cdcl$_3$) δ = 171.36, 135.95, 131.29, 125.06, 123.20, 121.59, 118.68, 118.11, 116.63, 33.87, 33.54, 27.71, 22.31, 9.63.
HRMS (ESI) calcd for $[M + H]^+$ ($C_{15}H_{20}ON$), 230.1539; found, 230.1538.

1-(3-Methyl-1*H*-indol-1-yl)-4-phenylbutan-1-one (3p)

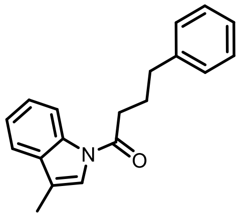

White solid, yield 93%.
^{1}H NMR (600 MHz, Chloroform-*d*) δ = 8.45 (s, 1H), 7.49 (d, *J* = 7.6, 1H), 7.38 – 7.27 (m, 4H), 7.26 – 7.21 (m, 3H), 7.11 (s, 1H), 2.86 (t, *J* = 7.4, 2H), 2.79 (t, *J* = 7.5, 2H), 2.26 (d, *J* = 1.1, 3H), 2.18 (p, J = 7.4 Hz, 2H).
^{13}C NMR (151 MHz, Chloroform-*d*) δ = 170.80, 141.23, 135.90, 131.31, 128.51, 128.46, 126.08, 125.10, 123.27, 121.53, 118.72, 118.19, 116.61, 35.00, 34.84, 26.03, 9.64.

HRMS (ESI) calcd for $[M + H]^+$ ($C_{19}H_{20}ON$), 278.1539; found, 278.1538.

1-(3-Methyl-1*H*-indol-1-yl)-4-(*p*-tolyl)butan-1-one (3q)

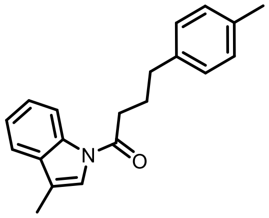

White solid, yield 76%
^{1}H NMR (600 MHz, Chloroform-*d*) δ = 8.46 (s, 1H), 7.51 – 7.47 (m, 1H), 7.38 – 7.23 (m, 3H), 7.17 – 7.07 (m, 4H), 2.91 – 2.80 (m, 2H), 2.77 – 2.72 (m, 2H), 2.39 – 2.30 (m, 3H), 2.30 – 2.23 (m, 3H), 2.19 – 2.13 (m, 2H).
^{13}C NMR (151 MHz, Chloroform-*d*) δ = 170.88, 138.11, 135.90, 135.53, 131.30, 129.13, 128.39, 125.08, 123.25, 121.57, 118.70, 118.14, 116.61, 34.84, 34.55, 26.12, 20.97, 9.64.

HRMS (ESI) calcd for $[M + H]^+$ ($C_{20}H_{22}ON$), 292.1696; found, 292.1694.

2-(Benzo[*d*][1,3]dioxol-5-yl)-1-(3-methyl-1*H*-indol-1-yl)ethan-1-one (3r)

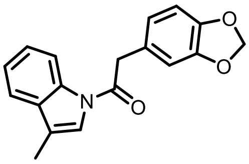

White solid, yield 85%

^{1}H NMR (600 MHz, dmso-*d6*) δ = 8.29 (d, *J* = 8.0, 1H), 7.78 (s, 1H), 7.53 (d, *J* = 7.5, 1H), 7.31 – 7.24 (m, 2H), 6.92 (d, *J* = 0.8, 1H), 6.85 (d, *J* = 7.9, 1H), 6.80 (d, *J* = 7.9, 1H), 5.97 (s, 2H), 4.23 (s, 2H), 2.23 (s, 3H).

^{13}C NMR (151 MHz, dmso-*d6*) δ = 170.22, 147.65, 146.57, 135.79, 131.53, 128.38, 125.19, 123.79, 123.06, 119.37, 117.52, 116.37, 110.44, 108.58, 101.30, 41.64, 9.78.

HRMS (ESI) calcd for $[M + Na]^+$ ($C_{18}H_{15}O_3NNa$), 316.0944; found, 316.0942.

(3-Methyl-1*H*-indol-1-yl)(*p*-tolyl)methanone (3s)[5]

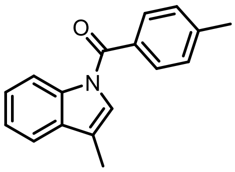

White solid, yield 93%

^{1}H NMR (600 MHz, Chloroform-*d*) δ = 8.36 (d, *J* = 8.2, 1H), 7.62 (d, *J* = 8.0, 2H), 7.53 (d, *J* = 7.8, 1H), 7.38 – 7.35 (m, 1H), 7.33 – 7.31 (m, 3H), 7.08 (d, *J* = 1.0, 1H), 2.45 (s, 3H), 2.25 (d, *J* = 1.2, 3H).

^{13}C NMR (151 MHz, Chloroform-*d*) δ = 168.35, 142.15, 136.34, 132.12, 131.75, 129.15, 129.10, 124.80, 124.46, 123.47, 118.74, 117.48, 116.39, 21.51, 9.55.

(3-Methyl-1*H*-indol-1-yl)(phenyl)methanone (3t)[6]

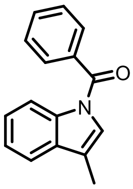

White solid, yield 96%

^{1}H NMR (600 MHz, Chloroform-*d*) δ = 8.41 (d, *J* = 8.1, 1H), 7.73 (d, *J* = 7.1, 2H), δ 7.60 (t, J = 7.4 Hz, 1H), 7.54 (q, J = 8.0, 7.4 Hz, 3H), 7.40 (t, J = 7.5 Hz, 1H), 7.35 (t, J = 7.3 Hz, 1H), 7.07 (s, 1H), 2.26 (s, 3H).

^{13}C NMR (151 MHz, Chloroform-*d*) δ = 168.34, 136.31, 135.00, 131.82, 131.56, 128.99, 128.48, 125.01, 124.33, 123.65, 118.82, 117.84, 116.50, 9.61.

1-(9*H*-carbazol-9-yl)butan-1-one (3u)[7]

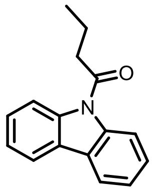

White solid, yield 84%

^{1}H NMR (600 MHz, Chloroform-d) δ 8.22 (d, J = 8.4 Hz, 2H), 7.98 (d, J = 7.6 Hz, 2H), 7.47 (t, J = 7.8 Hz, 2H), 7.37 (t, J = 7.4 Hz, 2H), 3.10 (t, J = 7.2 Hz, 1H), 1.97 (h, J = 7.3 Hz, 1H), 1.13 (t, J = 7.4 Hz, 1H).

^{13}C NMR (151 MHz, Chloroform-*d*) δ = 173.09, 138.58, 127.21, 126.37, 123.43, 119.72, 116.38, 41.00, 18.13, 13.77.

References

1 Thiel, V.; Brinkhoff, T.; Dickschat, J. S.; Wickel, S.; Grunenberg, J.; Wagner-Döbler, I.; Simon, M.; Schulz, S. *Org. Biomol. Chem.*, **2010**, *8*, 234-246.

2 Vargas, D. A.; Méndez, L.J.; Cánepa, A. S.; Bravo, R. D. Sulfated zirconia: an efficient and reusable heterogeneous catalyst in the friedel-crafts acylation reaction of 3-methylindole. *Catal Lett*. **2017**, *147*, 1496-1502.

3 Zhou, X. Y.; Chen, Xia. Na_2CO_3-catalyzed *N*-acylation of indoles with alkenyl carboxylates. *Synthesis*, **2019**, *51*, 516-521.

4 Zeng, J.; Zhang, Z. J.; Zhu, Q. Z.; Jiang, Z.Y.; Zhong, G. H. Simplification of natural β-carboline alkaloids to obtain indole derivatives as potent fungicides against rice sheath blight. *Molecules*, **2020**, *25*, 1189.

5 Kandukuri, S. R.; Oestreich, M. Aerobic palladium(II)-catalyzed dehydrogenation of cyclohexene-1-carbonyl indole amides: an indole-directed aromatization. *J. Org. Chem*., **2012**, *77*, 8750-8755.

6 García, B.; Ibeas, S.; Muñoz, M. S.; Navarro, A. M.; Peñacoba, I.; Leal, J. M. Protonation sites of indoles and benzoylindoles. *Eur. J.Org. Chem*. **2005**, *6*, 1161-1171.

7 Kolli, S. K.; Prasad, B.; Babu, P. V.; Ashfaq, M. A.; Ehtesham, N. Z.; Raju, R. R.; Pal, M. TFAA/H_3PO_4 mediated unprecedented *N*-acylation of carbazoles leading to small molecules possessing anti-proliferative activities against cancer cells. *Org. Biomol. Chem.*, **2014**, *12*, 6080-6084.

NMR spectra

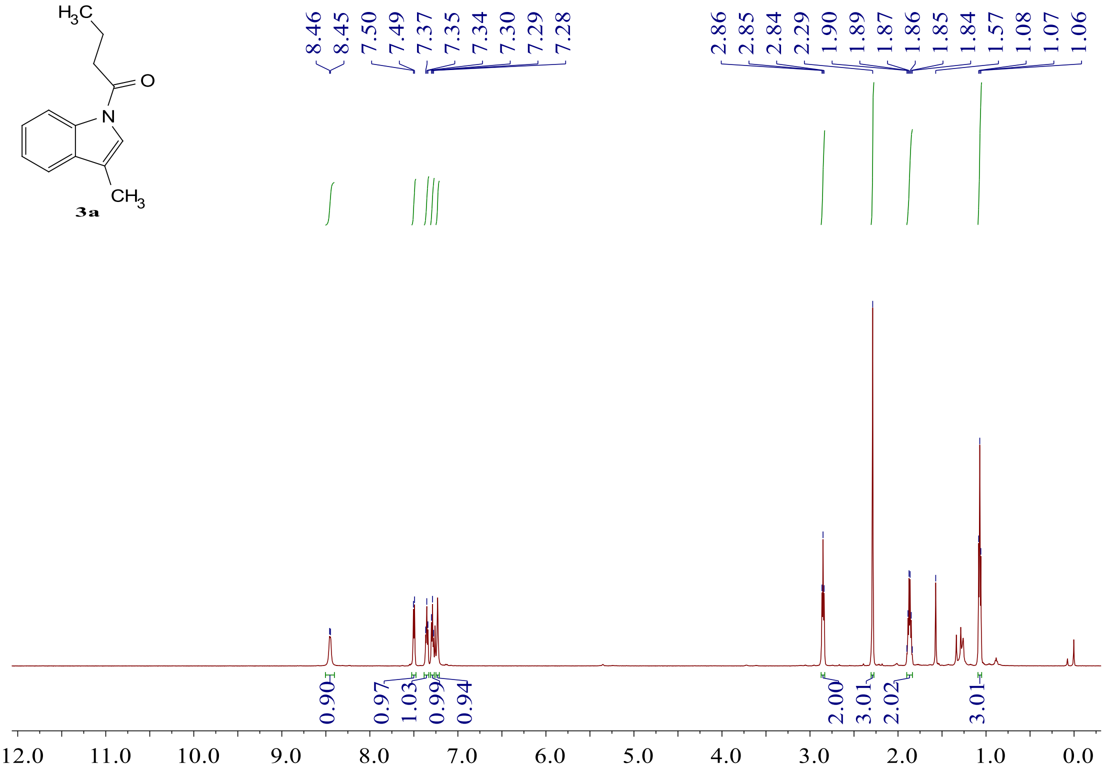
H3C
O
N
CH3
3a
8.46
8.45
7.50
7.49
7.37
7.35
7.34
7.30
7.29
7.28
2.86
2.85
2.84
2.29
1.90
1.89
1.87
1.86
1.85
1.84
1.57
1.08
1.07
1.06
0.90
0.97
1.03
0.99
0.94
2.00
3.01
2.02
3.01
12.0 11.0 10.0 9.0 8.0 7.0 6.0 5.0 4.0 3.0 2.0 1.0 0.0

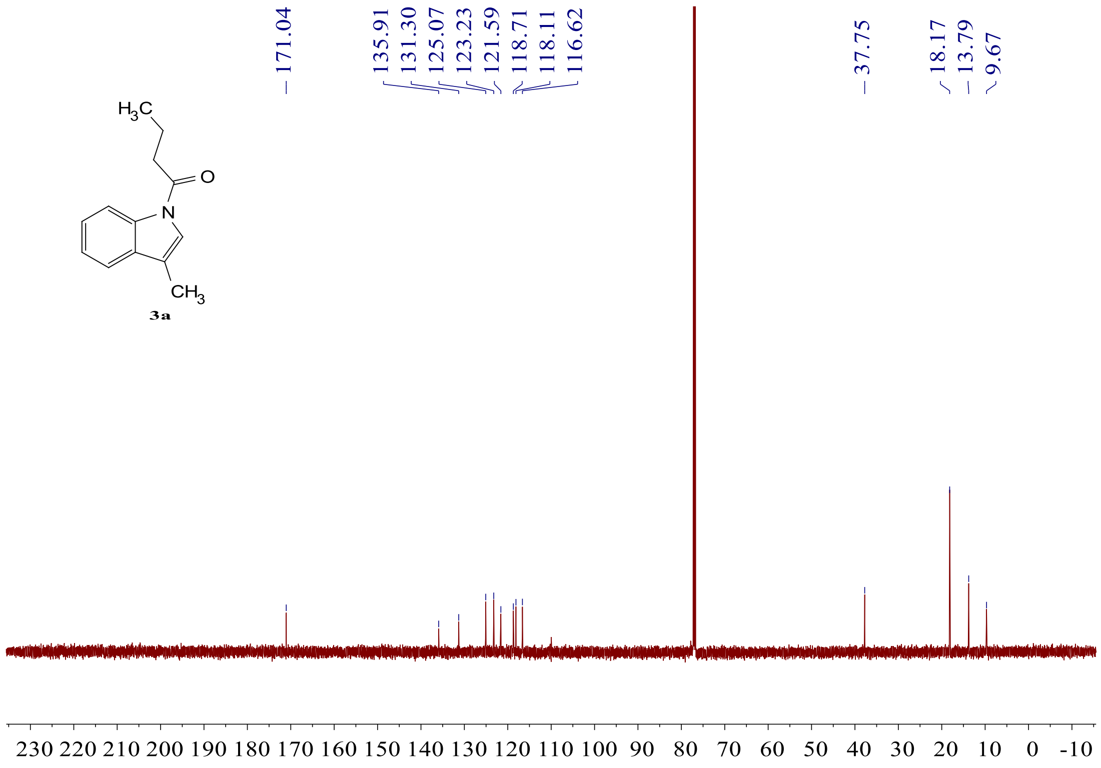
171.04
135.91
131.30
125.07
123.23
121.59
118.71
118.11
116.62
37.75
18.17
13.79
9.67
H3C
O
N
CH3
3a
230 220 210 200 190 180 170 160 150 140 130 120 110 100 90 80 70 60 50 40 30 20 10 0 -10

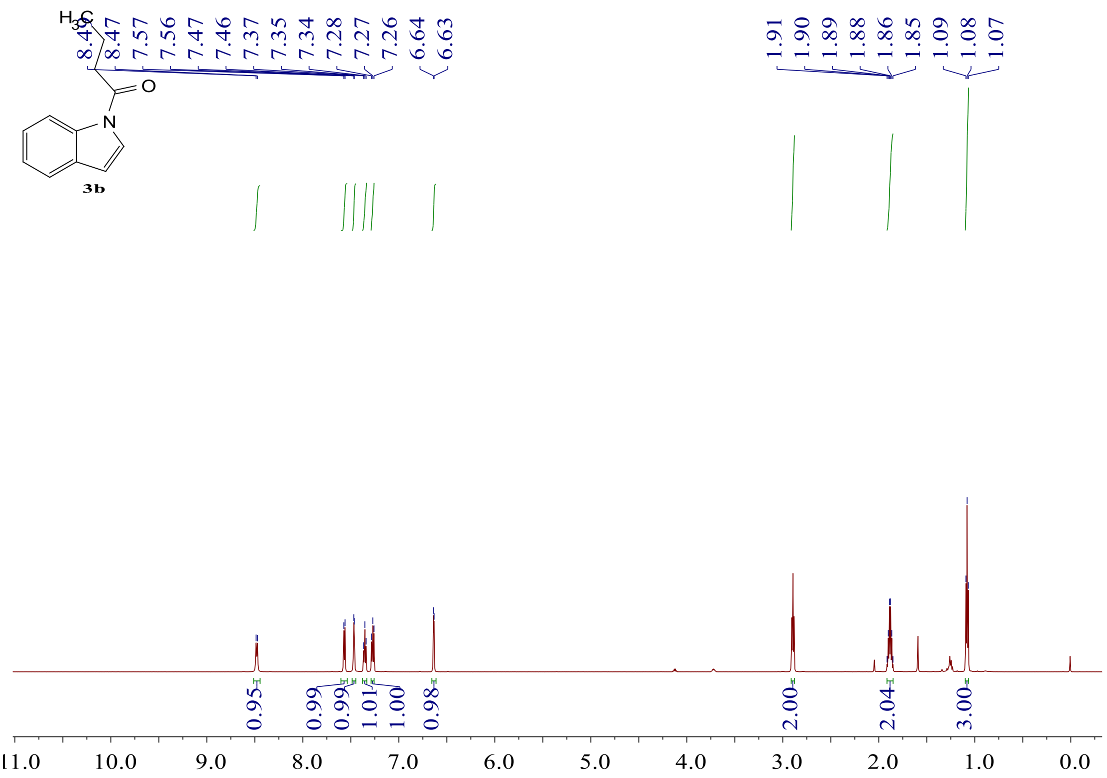
3b
8.48
8.47
7.57
7.56
7.47
7.46
7.37
7.35
7.34
7.28
7.27
7.26
6.64
6.63
1.91
1.90
1.89
1.88
1.86
1.85
1.09
1.08
1.07
0.95
0.99
0.99
1.01
1.00
0.98
2.00
2.04
3.00
11.0 10.0 9.0 8.0 7.0 6.0 5.0 4.0 3.0 2.0 1.0 0.0

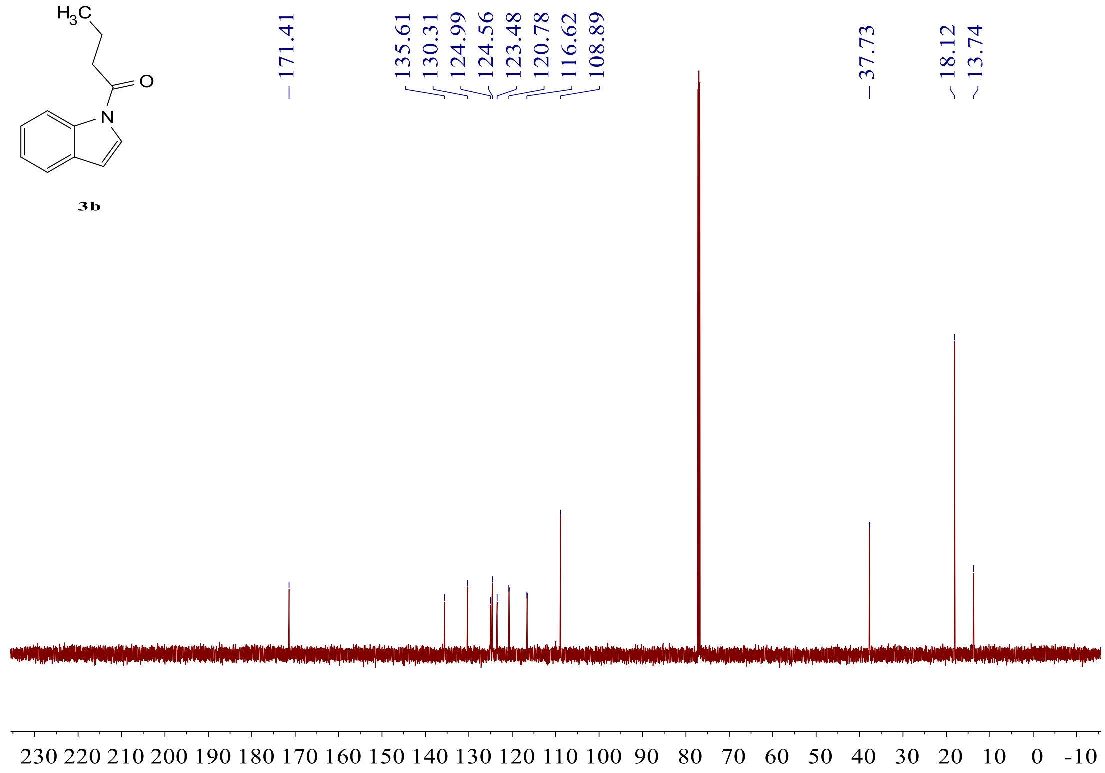
3b
171.41
135.61
130.31
124.99
124.56
123.48
120.78
116.62
108.89
37.73
18.12
13.74
230 220 210 200 190 180 170 160 150 140 130 120 110 100 90 80 70 60 50 40 30 20 10 0 -10

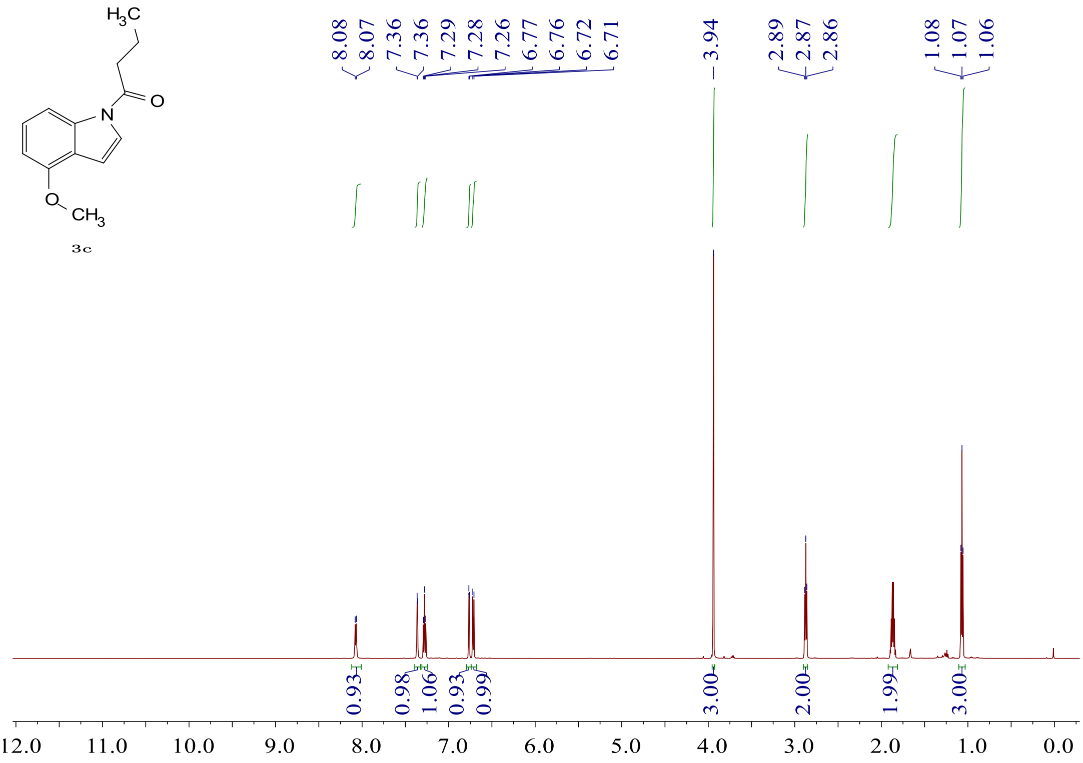
H3C
N
O
O
CH3
3c
8.08
8.07
7.36
7.36
7.29
7.28
7.26
6.77
6.76
6.72
6.71
3.94
2.89
2.87
2.86
1.08
1.07
1.06
0.93
0.98
1.06
0.93
0.99
3.00
2.00
1.99
3.00
12.0 11.0 10.0 9.0 8.0 7.0 6.0 5.0 4.0 3.0 2.0 1.0 0.0

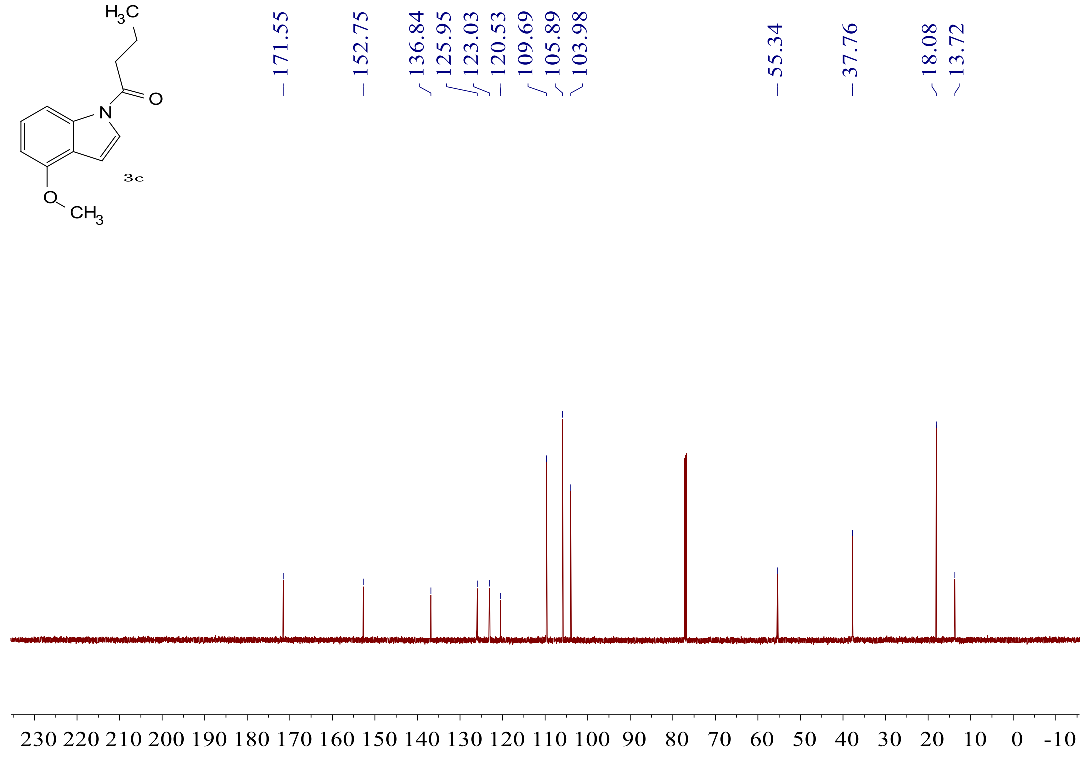
H3C
N
O
3c
O
CH3
171.55
152.75
136.84
125.95
123.03
120.53
109.69
105.89
103.98
55.34
37.76
18.08
13.72
230 220 210 200 190 180 170 160 150 140 130 120 110 100 90 80 70 60 50 40 30 20 10 0 -10

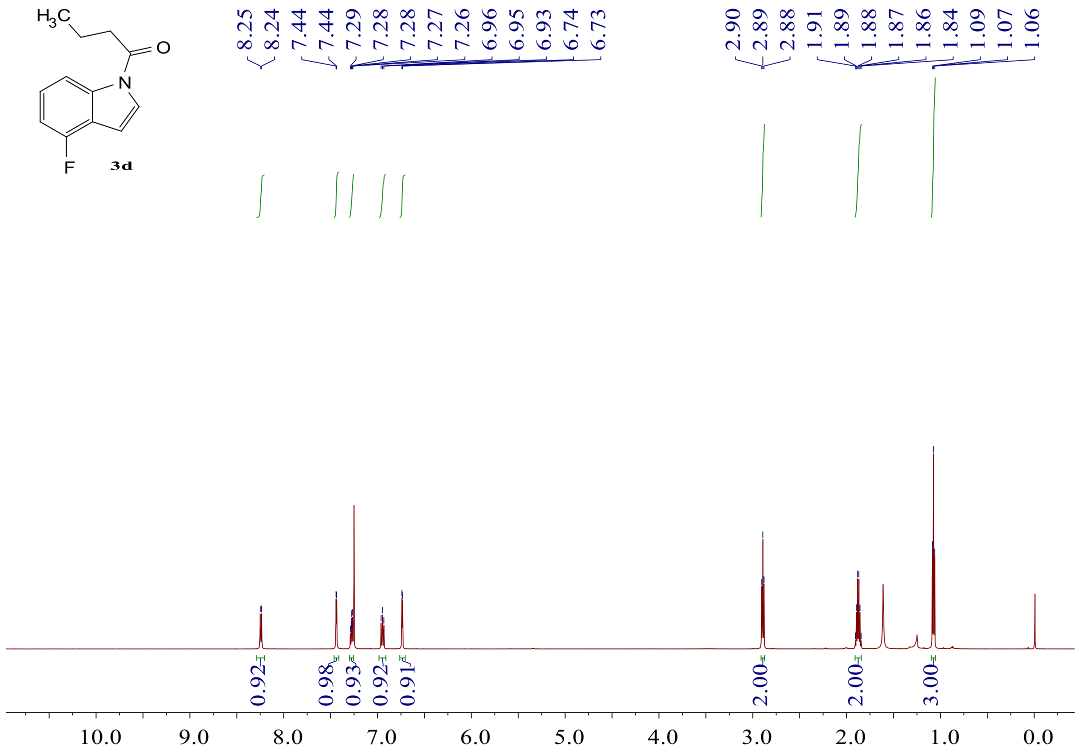
H3C
O
N
F
3d
8.25
8.24
7.44
7.44
7.29
7.28
7.28
7.27
7.26
6.96
6.95
6.93
6.74
6.73
2.90
2.89
2.88
1.91
1.89
1.88
1.87
1.86
1.84
1.09
1.07
1.06
0.92
0.98
0.93
0.92
0.91
2.00
2.00
3.00
10.0
9.0
8.0
7.0
6.0
5.0
4.0
3.0
2.0
1.0
0.0

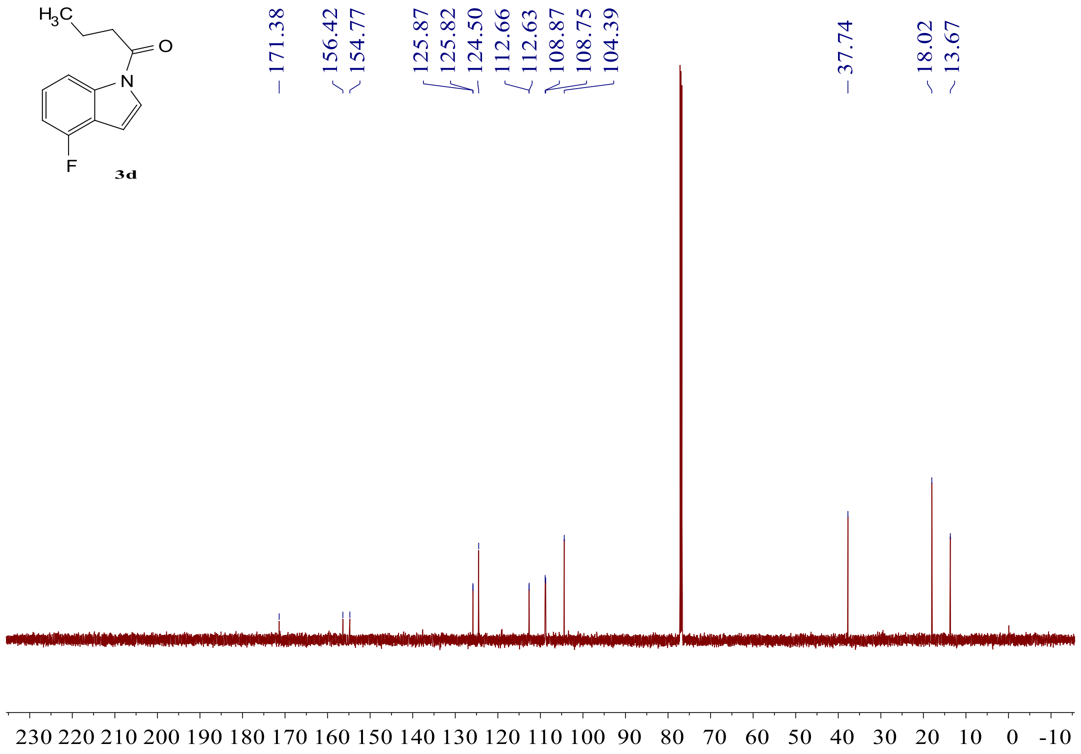
H3C
O
N
F
3d
171.38
156.42
154.77
125.87
125.82
124.50
112.66
112.63
108.87
108.75
104.39
37.74
18.02
13.67
230 220 210 200 190 180 170 160 150 140 130 120 110 100 90 80 70 60 50 40 30 20 10 0 -10

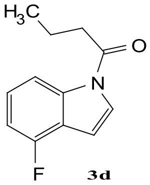
H3C
O
N
F

3d

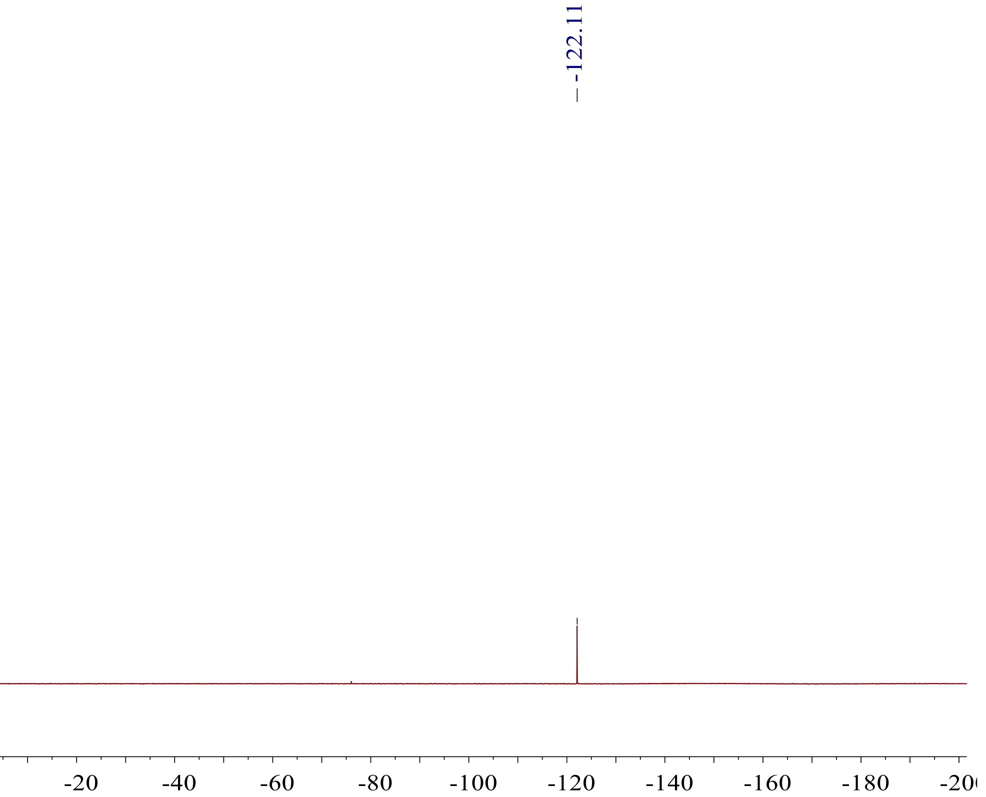
-122.11
20 0 -20 -40 -60 -80 -100 -120 -140 -160 -180

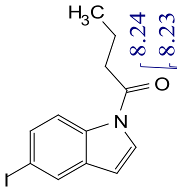
H3C
O
N
I

3e

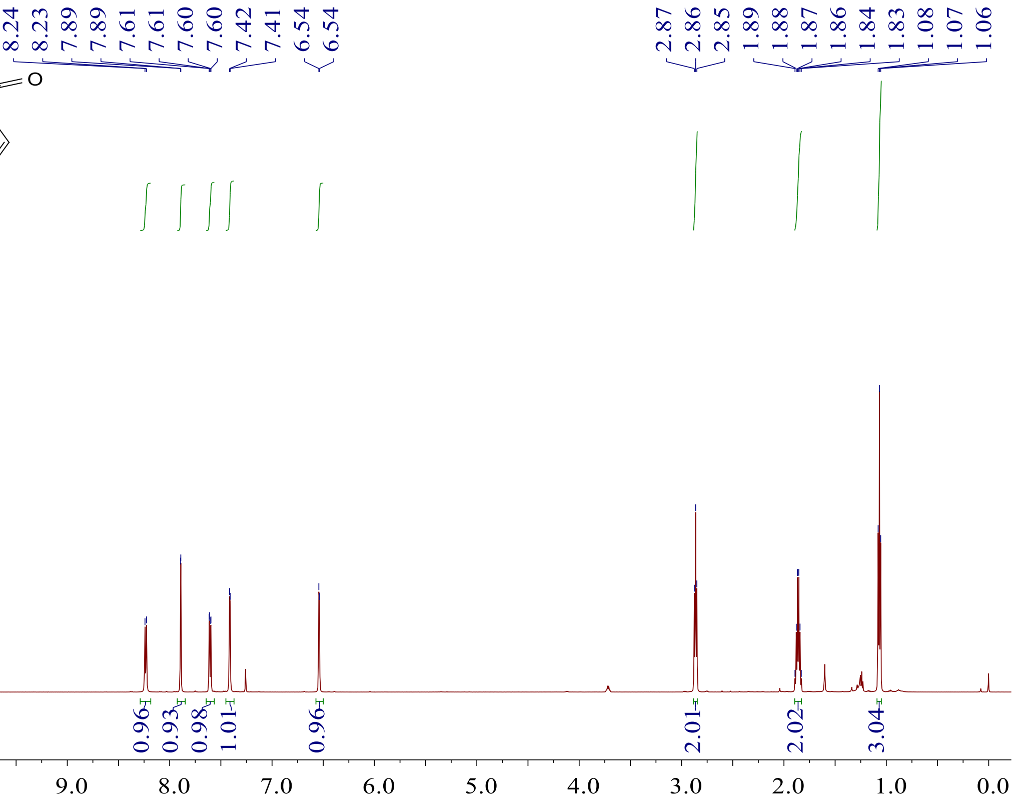
8.24 8.23 7.89 7.89 7.61 7.61 7.60 7.60 7.42 7.41 6.54 6.54
2.87 2.86 2.85 1.89 1.88 1.87 1.86 1.84 1.83 1.08 1.07 1.06
0.96 0.93 0.98 1.01 0.96 2.01 2.02 3.04
10.0 9.0 8.0 7.0 6.0 5.0 4.0 3.0 2.0 1.0 0.0

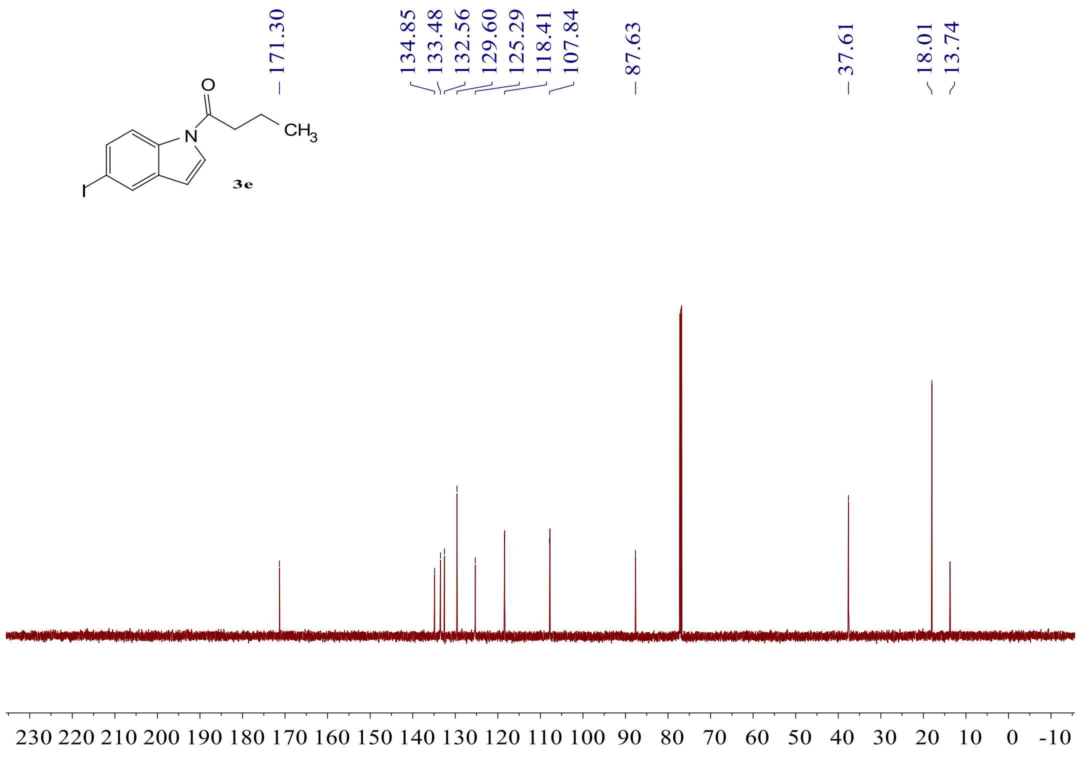
171.30
134.85
133.48
132.56
129.60
125.29
118.41
107.84
87.63
37.61
18.01
13.74
3e
230 220 210 200 190 180 170 160 150 140 130 120 110 100 90 80 70 60 50 40 30 20 10 0 -10

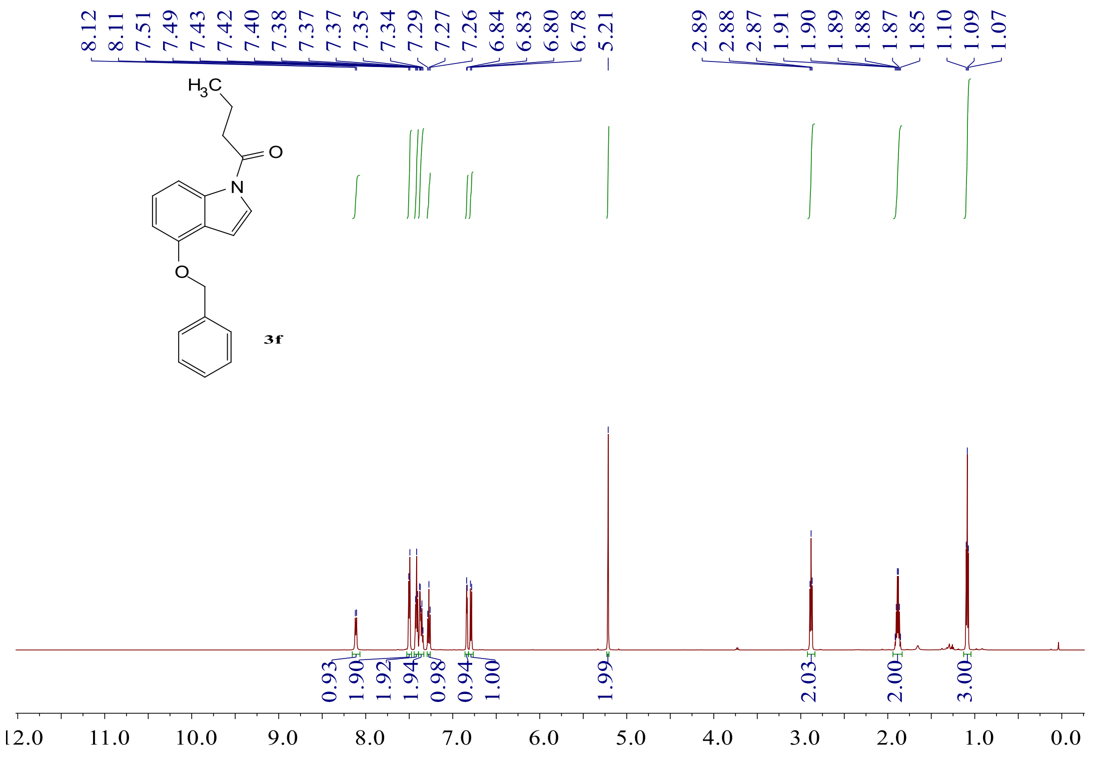
8.12
8.11
7.51
7.49
7.43
7.42
7.40
7.38
7.37
7.37
7.35
7.34
7.29
7.27
7.26
6.84
6.83
6.80
6.78
5.21
2.89
2.88
2.87
1.91
1.90
1.89
1.88
1.87
1.85
1.10
1.09
1.07
3f
0.93
1.90
1.92
1.94
0.98
0.94
1.00
1.99
2.03
2.00
3.00
12.0 11.0 10.0 9.0 8.0 7.0 6.0 5.0 4.0 3.0 2.0 1.0 0.0

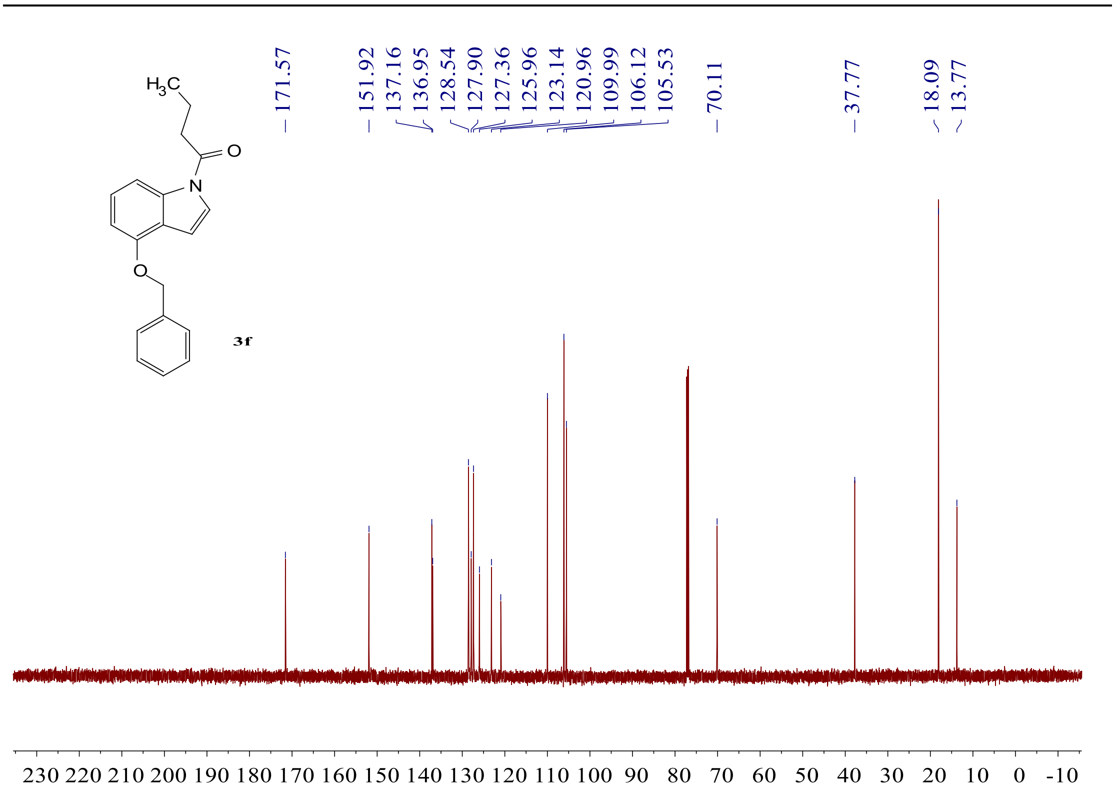
171.57
151.92
137.16
136.95
128.54
127.90
127.36
125.96
123.14
120.96
109.99
106.12
105.53
70.11
37.77
18.09
13.77
3f
230 220 210 200 190 180 170 160 150 140 130 120 110 100 90 80 70 60 50 40 30 20 10 0 -10

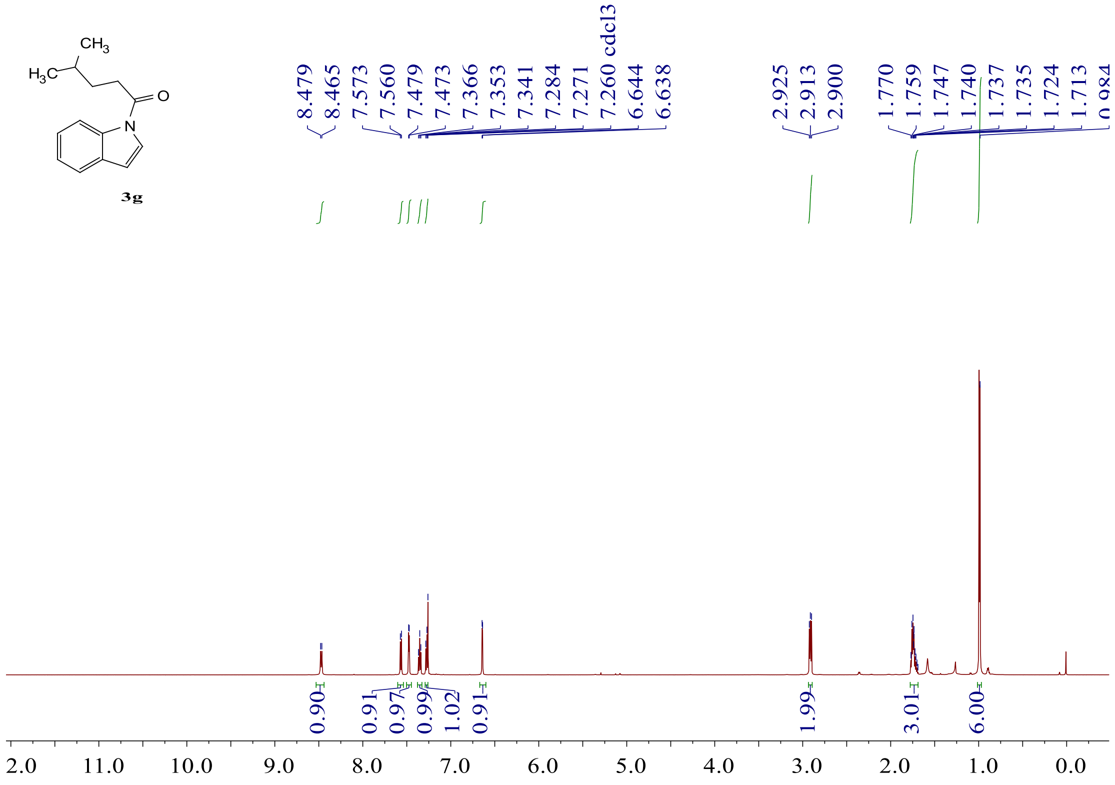
8.479
8.465
7.573
7.560
7.479
7.473
7.366
7.353
7.341
7.284
7.271
7.260 cdcl3
6.644
6.638
2.925
2.913
2.900
1.770
1.759
1.747
1.740
1.737
1.735
1.724
1.713
3g
0.90
0.91
0.97
0.99
1.02
0.91
1.99
3.01
6.00
2.0 11.0 10.0 9.0 8.0 7.0 6.0 5.0 4.0 3.0 2.0 1.0 0.0

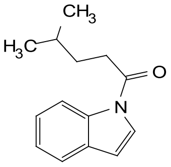

3g

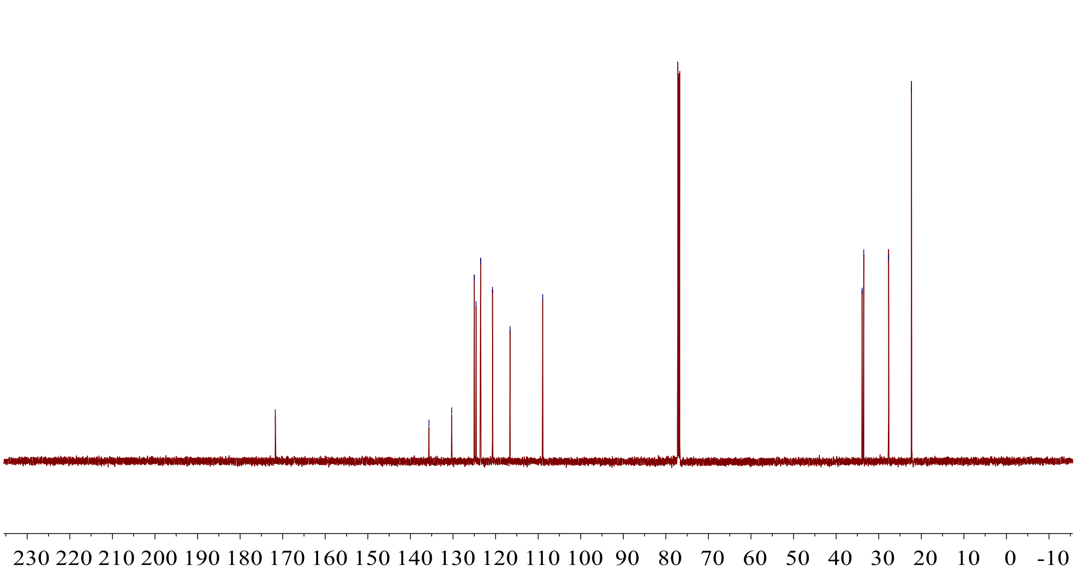
171.73
135.64
130.31
125.03
124.59
123.49
120.72
116.60
108.91
33.90
33.51
27.68
22.30
230 220 210 200 190 180 170 160 150 140 130 120 110 100 90 80 70 60 50 40 30 20 10 0 -10

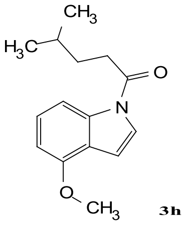

3h

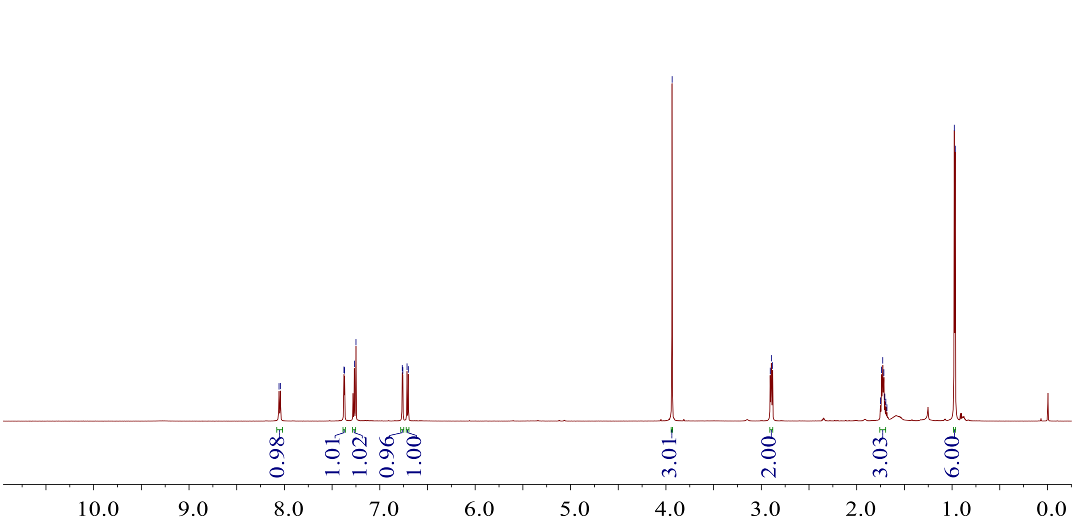
8.06
8.04
7.38
7.37
7.27
7.25
6.76
6.76
6.71
6.70
3.93
2.91
2.90
2.88
1.75
1.74
1.73
1.72
1.71
1.69
1.68
0.98
0.97
0.98
1.01
1.02
0.96
1.00
3.01
2.00
3.03
6.00
10.0 9.0 8.0 7.0 6.0 5.0 4.0 3.0 2.0 1.0 0.0

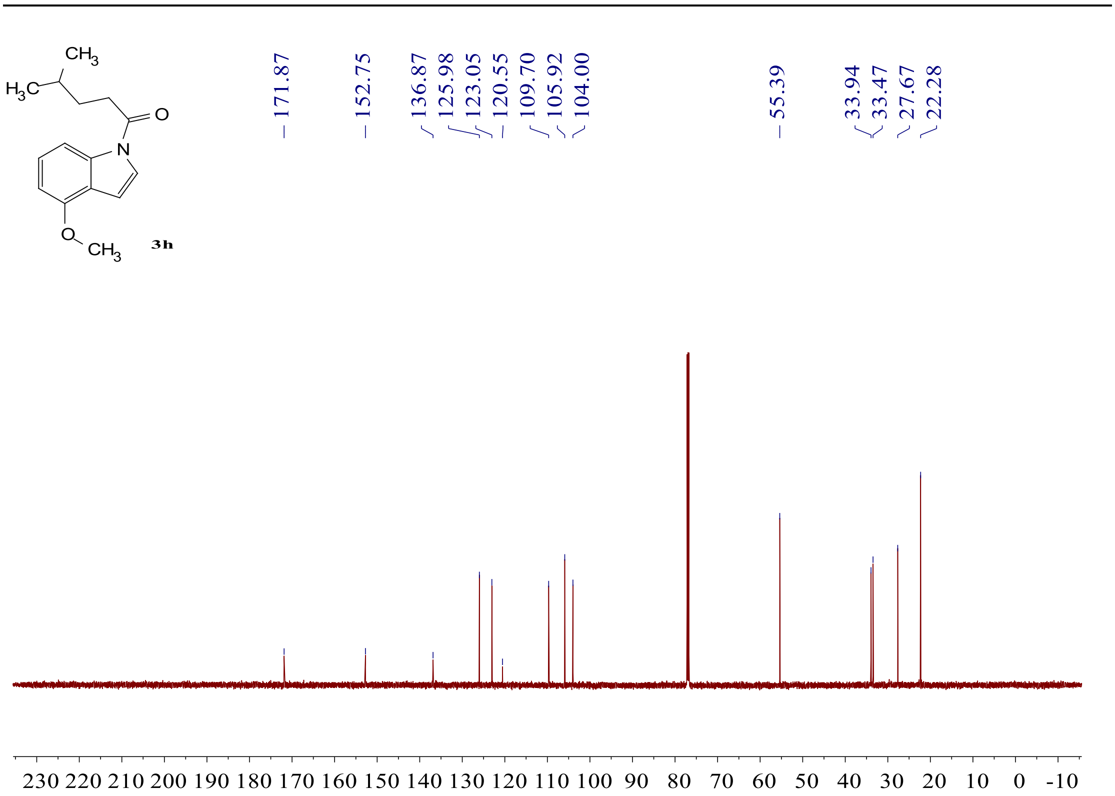
3h
171.87
152.75
136.87
125.98
123.05
120.55
109.70
105.92
104.00
55.39
33.94
33.47
27.67
22.28
230 220 210 200 190 180 170 160 150 140 130 120 110 100 90 80 70 60 50 40 30 20 10 0 -10

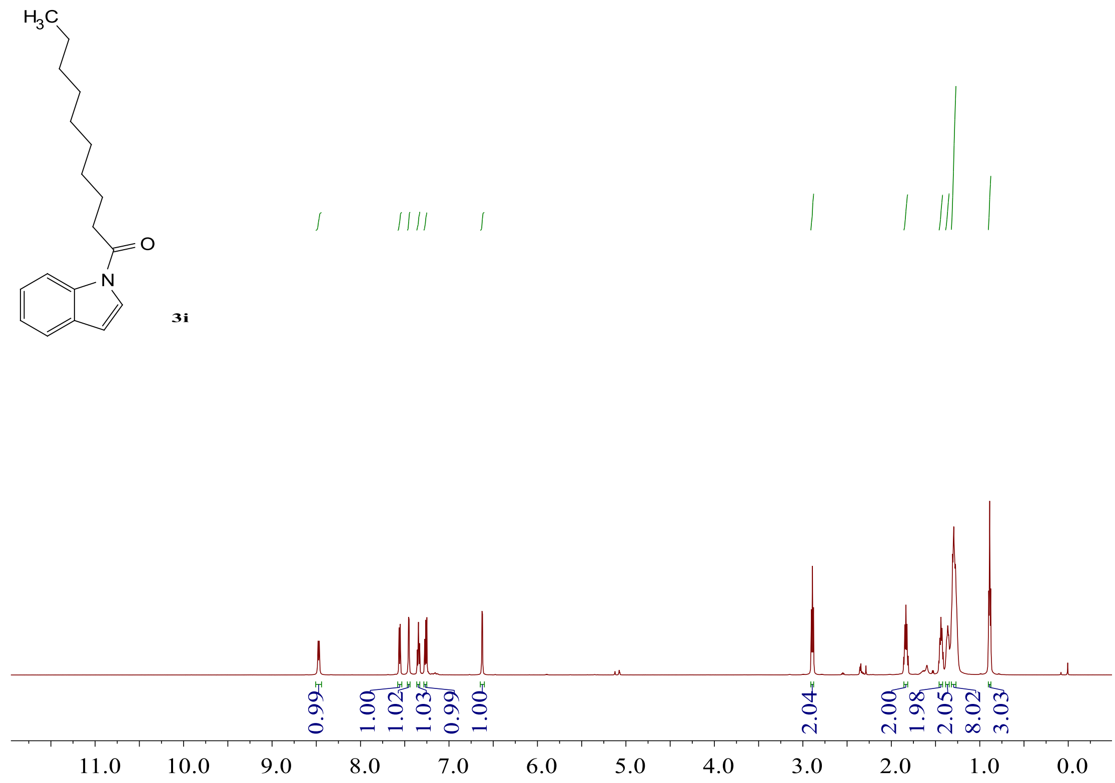
3i
0.99
1.00
1.02
1.03
0.99
1.00
2.04
2.00
1.98
2.05
8.02
3.03
11.0 10.0 9.0 8.0 7.0 6.0 5.0 4.0 3.0 2.0 1.0 0.0

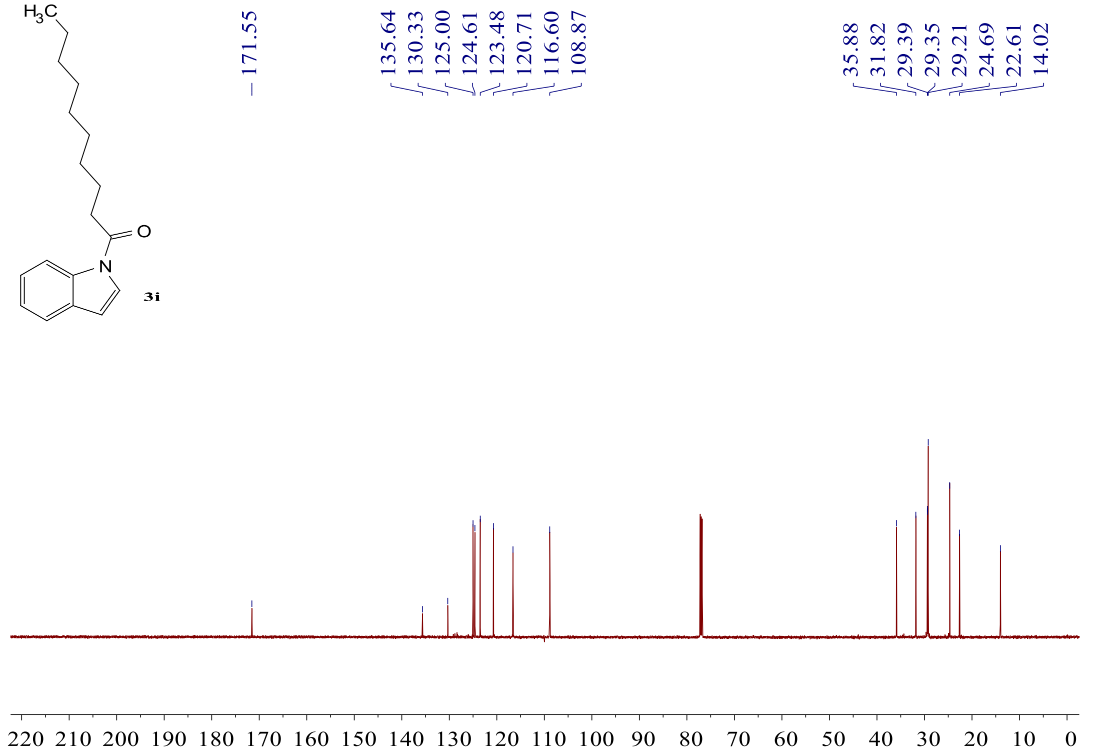
H3C
O
N
171.55
135.64
130.33
125.00
124.61
123.48
120.71
116.60
108.87
35.88
31.82
29.39
29.35
29.21
24.69
22.61
14.02
220 210 200 190 180 170 160 150 140 130 120 110 100 90 80 70 60 50 40 30 20 10 0

3i

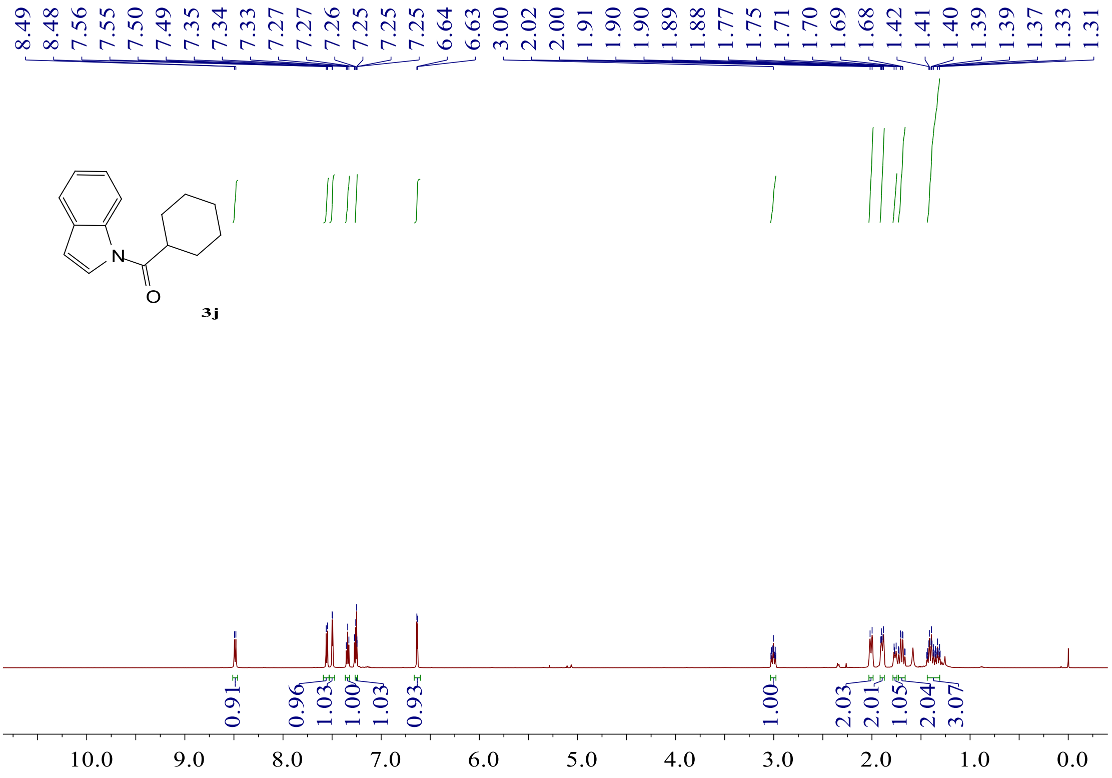
N
O
8.49
8.48
7.56
7.55
7.50
7.49
7.35
7.34
7.33
7.27
7.27
7.26
7.25
7.25
7.25
6.64
6.63
3.00
2.02
2.00
1.91
1.90
1.90
1.89
1.88
1.77
1.75
1.71
1.70
1.69
1.68
1.42
1.41
1.40
1.39
1.39
1.37
1.33
1.31
0.91
0.96
1.03
1.00
1.03
0.93
1.00
2.03
2.01
1.05
2.04
3.07
10.0 9.0 8.0 7.0 6.0 5.0 4.0 3.0 2.0 1.0 0.0

3j

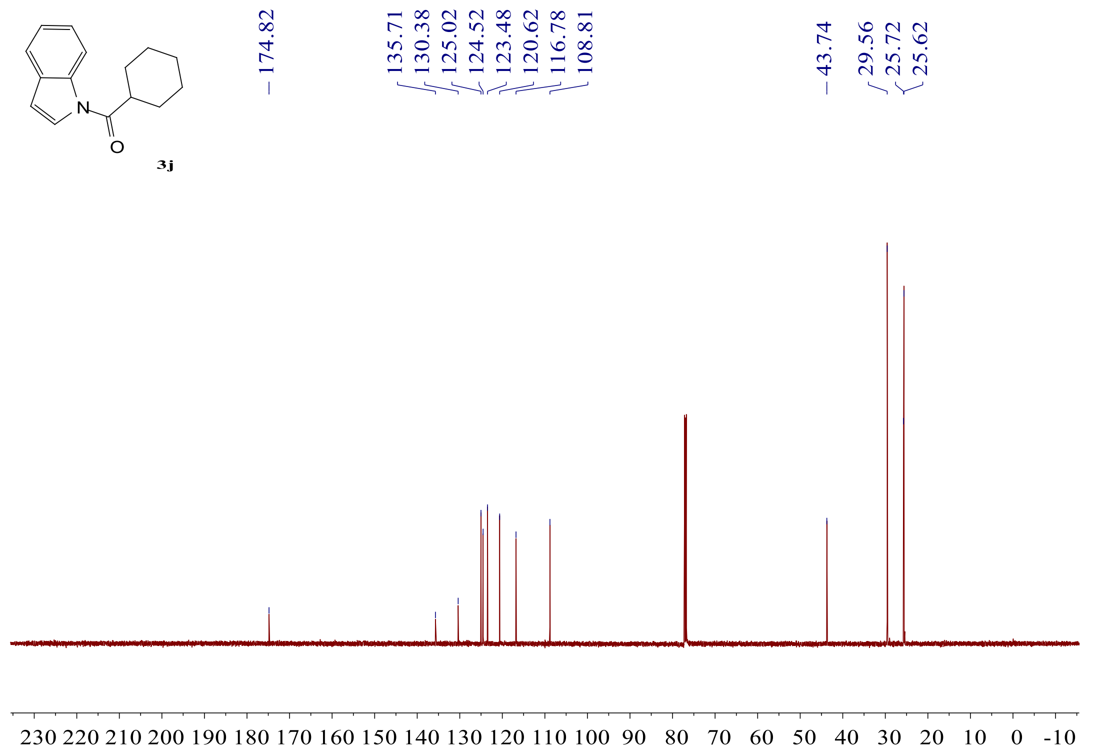
N
O
3j
174.82
135.71
130.38
125.02
124.52
123.48
120.62
116.78
108.81
43.74
29.56
25.72
25.62
230 220 210 200 190 180 170 160 150 140 130 120 110 100 90 80 70 60 50 40 30 20 10 0 -10

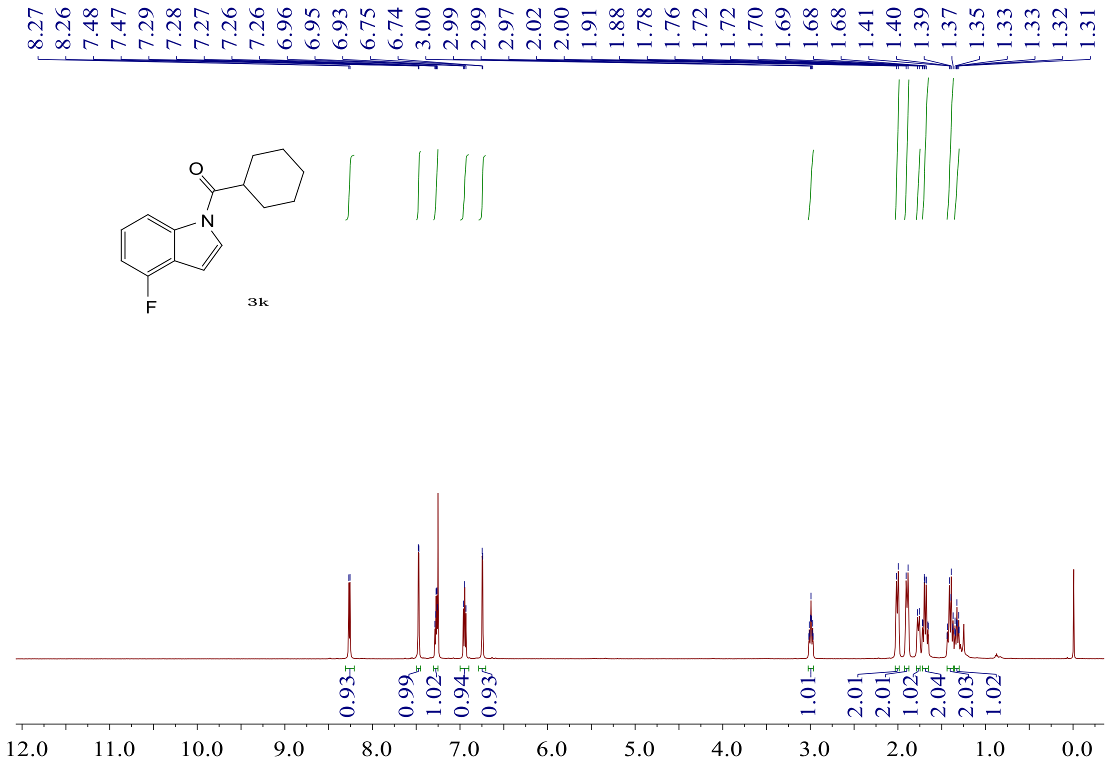
8.27
8.26
7.48
7.47
7.29
7.28
7.27
7.26
7.26
6.96
6.95
6.93
6.75
6.74
3.00
2.99
2.99
2.97
2.02
2.00
1.91
1.88
1.78
1.76
1.72
1.72
1.70
1.69
1.68
1.68
1.41
1.40
1.39
1.37
1.35
1.33
1.33
1.32
1.31
O
N
F
3k
0.93
0.99
1.02
0.94
0.93
1.01
2.01
2.01
1.02
2.04
2.03
1.02
12.0 11.0 10.0 9.0 8.0 7.0 6.0 5.0 4.0 3.0 2.0 1.0 0.0

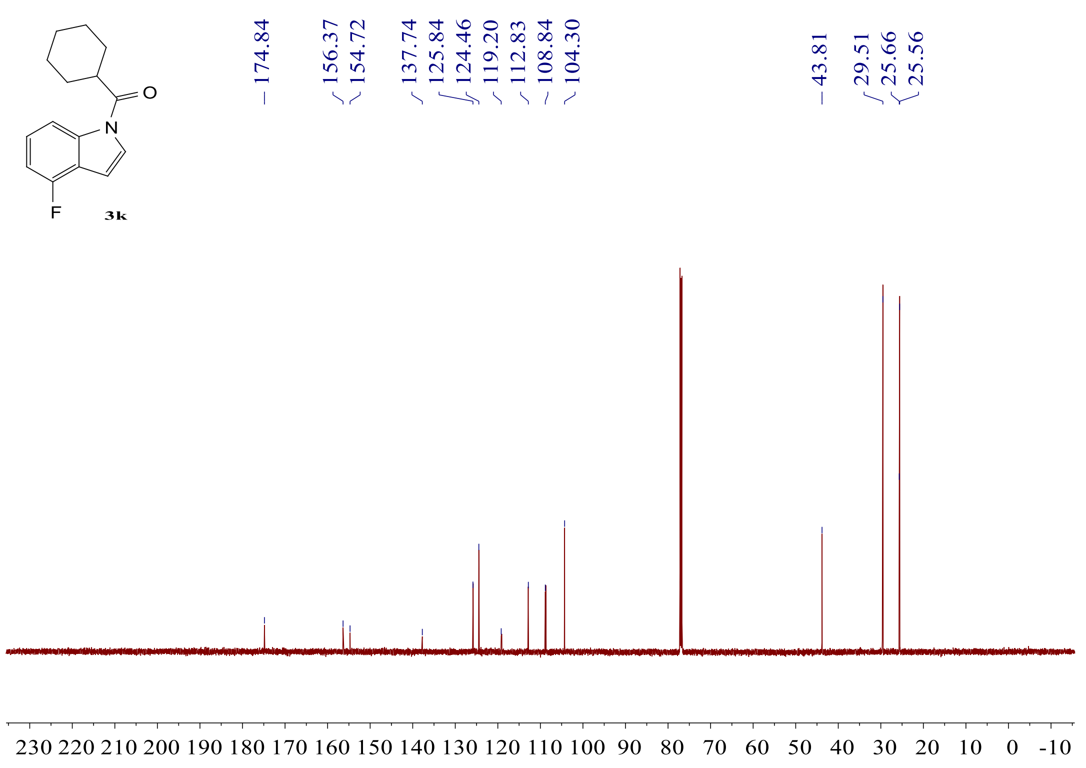
3k
F
N
O
174.84
156.37
154.72
137.74
125.84
124.46
119.20
112.83
108.84
104.30
43.81
29.51
25.66
25.56
230 220 210 200 190 180 170 160 150 140 130 120 110 100 90 80 70 60 50 40 30 20 10 0 -10

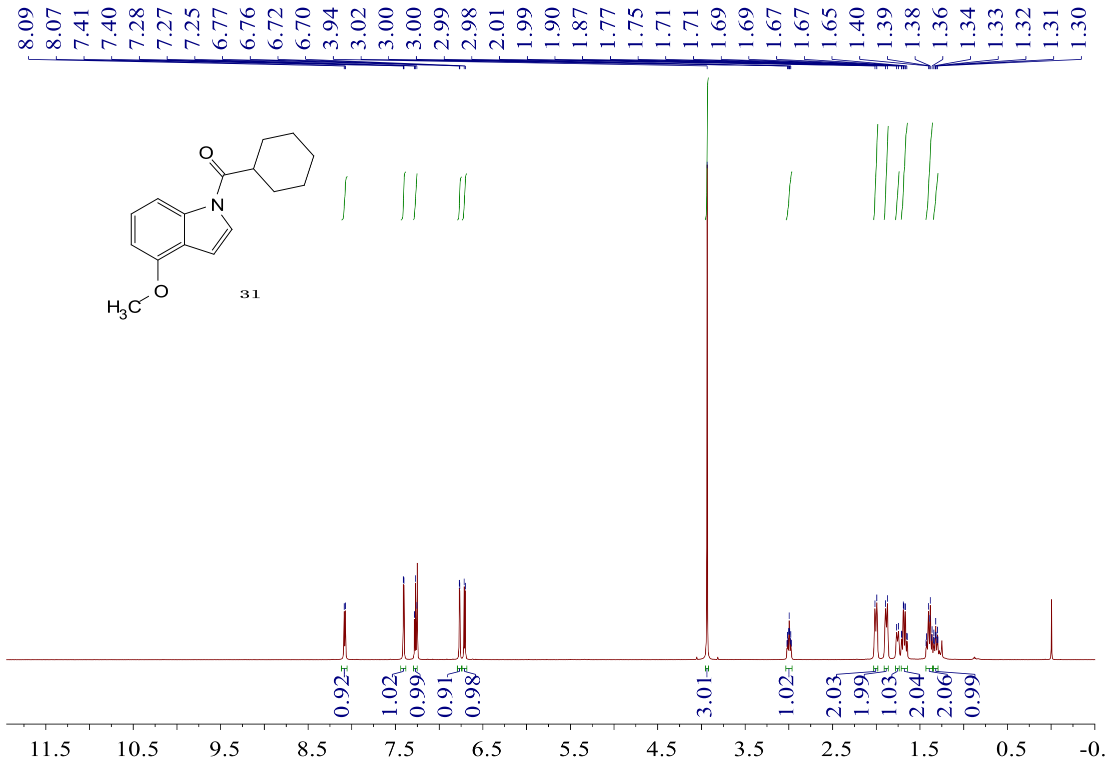
8.09
8.07
7.41
7.40
7.28
7.27
7.25
6.77
6.76
6.72
6.70
3.94
3.02
3.00
3.00
2.99
2.98
2.01
1.99
1.90
1.87
1.77
1.75
1.71
1.71
1.69
1.69
1.67
1.67
1.65
1.40
1.39
1.38
1.36
1.34
1.33
1.32
1.31
1.30
O
N
H3C
31
0.92
1.02
0.99
0.91
0.98
3.01
1.02
2.03
1.99
1.03
2.04
2.06
0.99
11.5 10.5 9.5 8.5 7.5 6.5 5.5 4.5 3.5 2.5 1.5 0.5 -0.

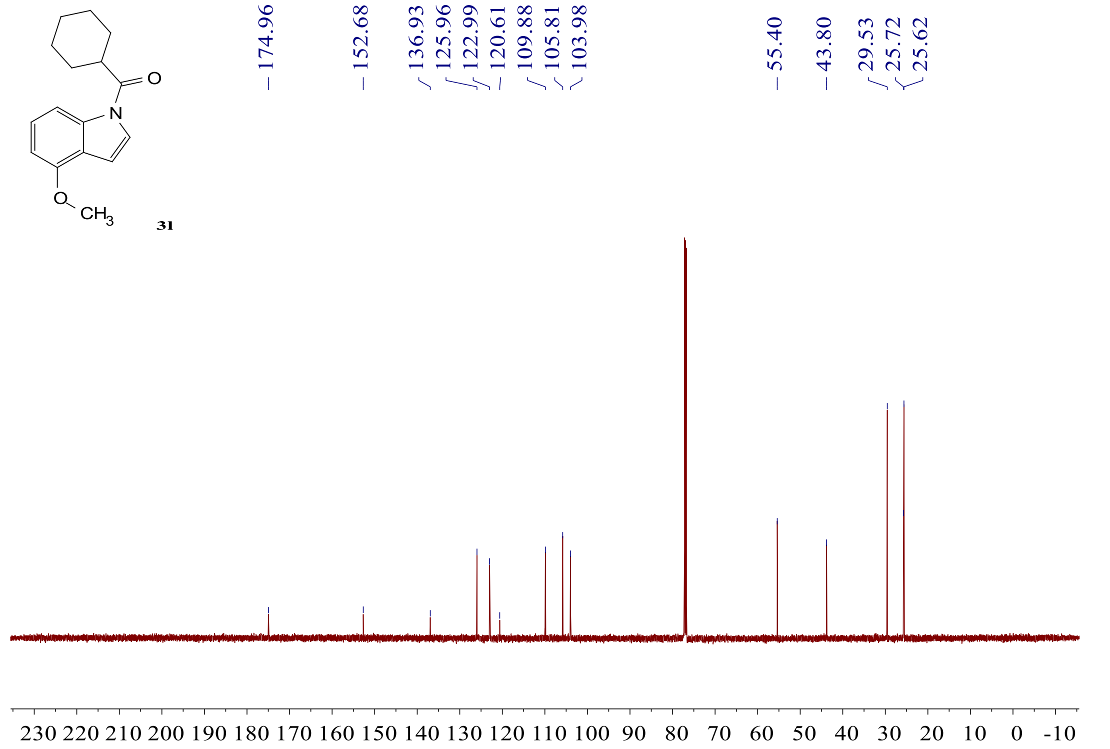
174.96
152.68
136.93
125.96
122.99
120.61
109.88
105.81
103.98
55.40
43.80
29.53
25.72
25.62
N
O
O
CH3
230 220 210 200 190 180 170 160 150 140 130 120 110 100 90 80 70 60 50 40 30 20 10 0 -10

3l

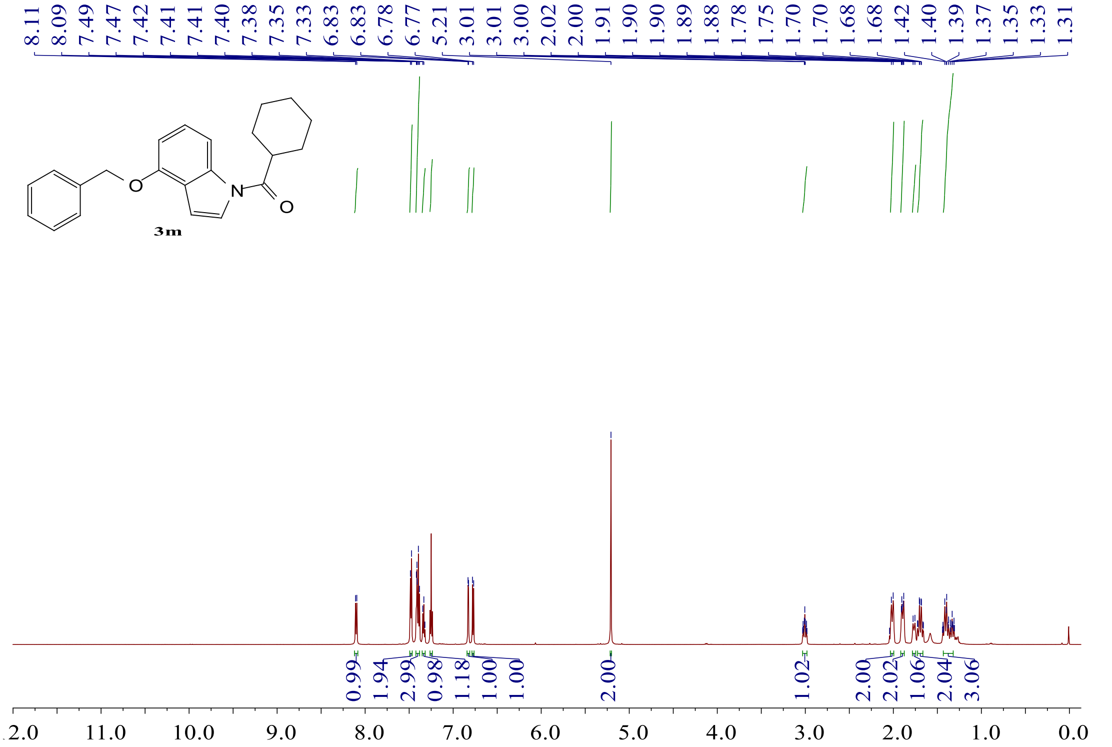
8.11
8.09
7.49
7.47
7.42
7.41
7.41
7.40
7.38
7.35
7.33
6.83
6.83
6.78
6.77
5.21
3.01
3.01
3.00
2.02
2.00
1.91
1.90
1.90
1.89
1.88
1.78
1.75
1.70
1.70
1.68
1.68
1.42
1.40
1.39
1.37
1.35
1.33
1.31
O
N
O
0.99
1.94
2.99
0.98
1.18
1.00
1.00
2.00
1.02
2.00
2.02
1.06
2.04
3.06
12.0 11.0 10.0 9.0 8.0 7.0 6.0 5.0 4.0 3.0 2.0 1.0 0.0

3m

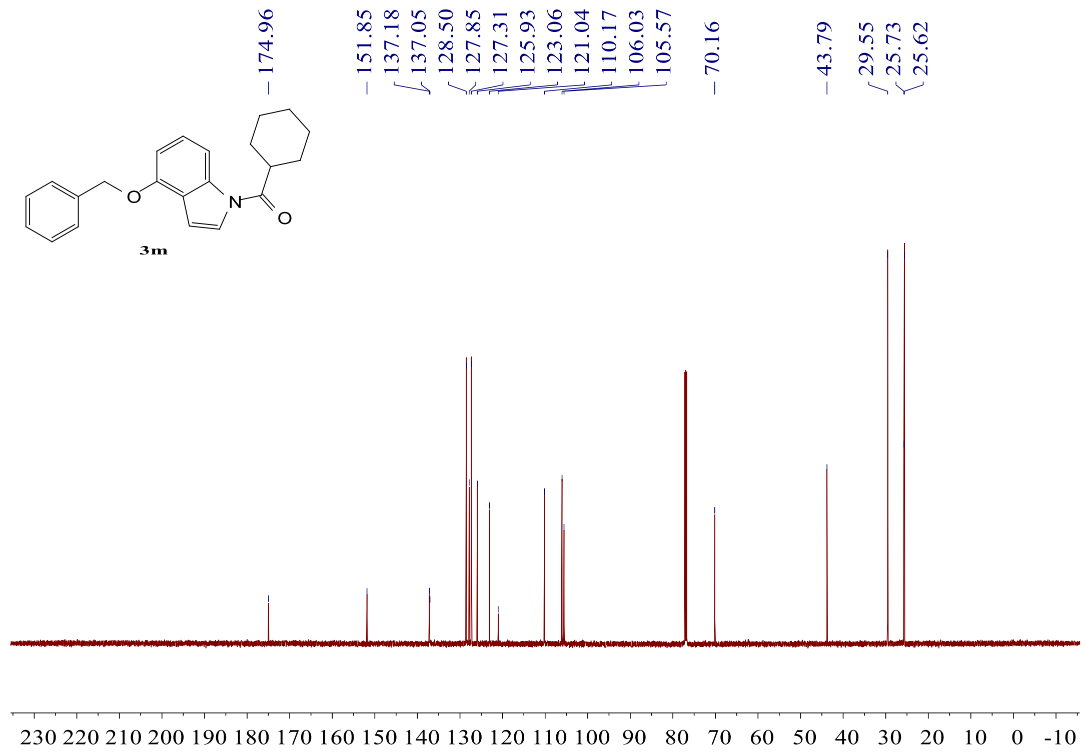
174.96
151.85
137.18
137.05
128.50
127.85
127.31
125.93
123.06
121.04
110.17
106.03
105.57
70.16
43.79
29.55
25.73
25.62
N
O
3m
230 220 210 200 190 180 170 160 150 140 130 120 110 100 90 80 70 60 50 40 30 20 10 0 -10

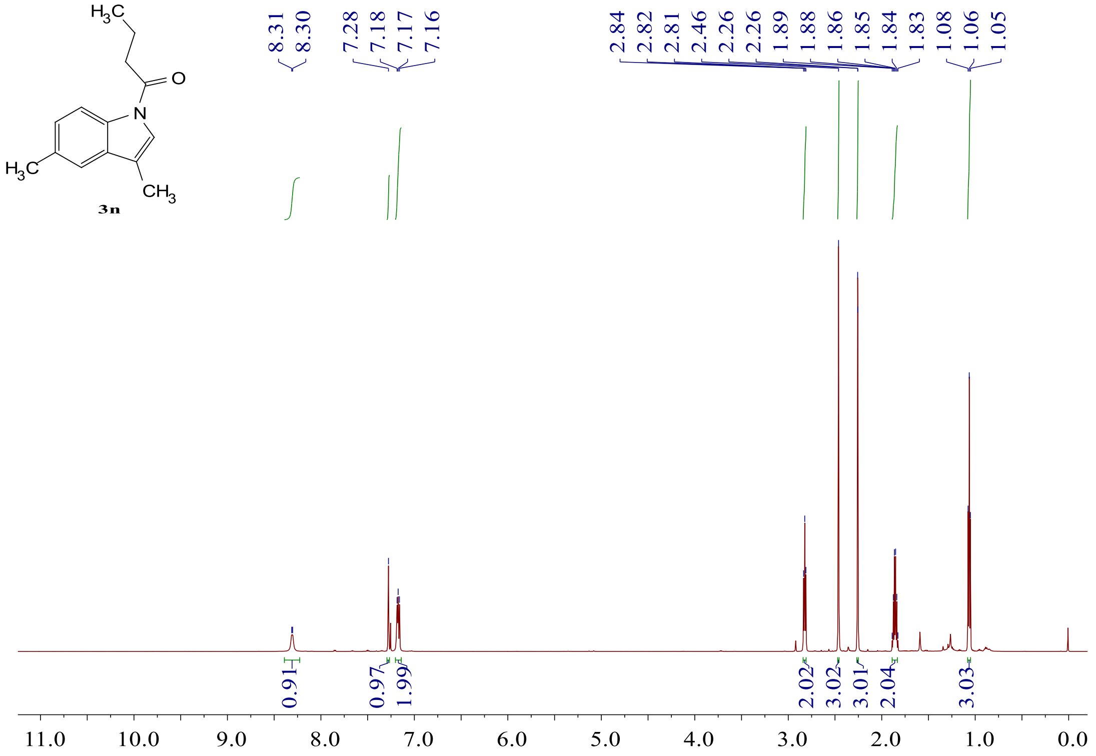
H3C
O
N
H3C
CH3
3n
8.31
8.30
7.28
7.18
7.17
7.16
2.84
2.82
2.81
2.46
2.26
2.26
1.89
1.88
1.86
1.85
1.84
1.83
1.08
1.06
1.05
0.91
0.97
1.99
2.02
3.02
3.01
2.04
3.03
11.0 10.0 9.0 8.0 7.0 6.0 5.0 4.0 3.0 2.0 1.0 0.0

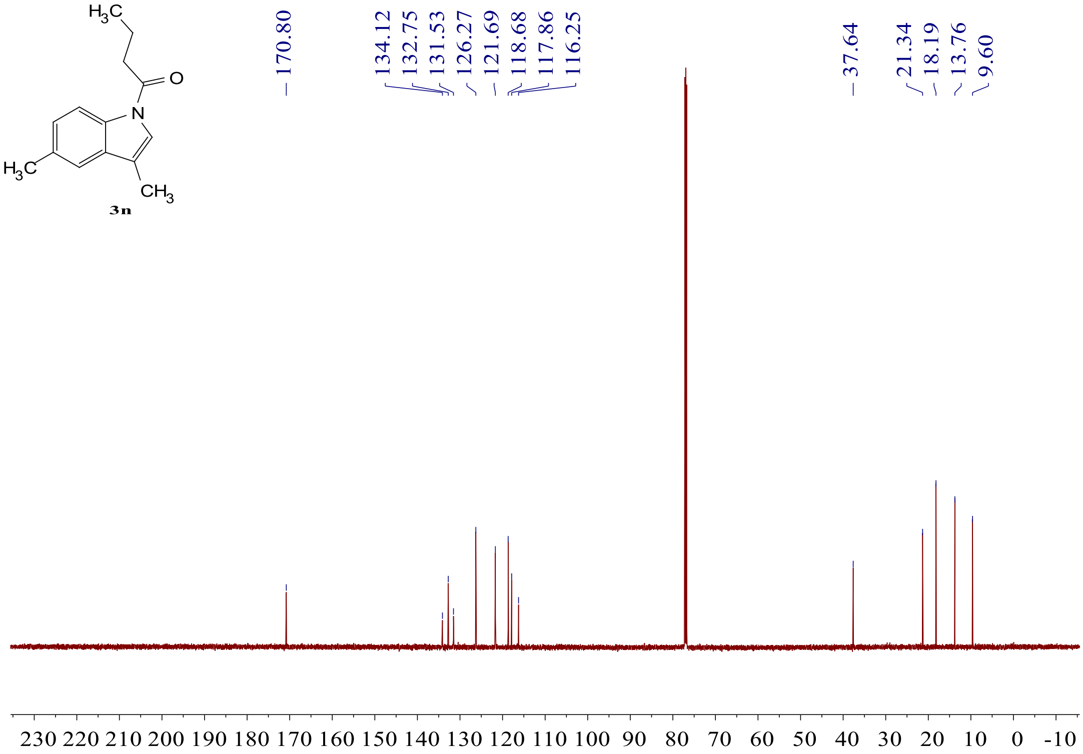
H3C
O
N
H3C
CH3
3n
170.80
134.12 132.75 131.53 126.27 121.69 118.68 117.86 116.25
37.64
21.34 18.19 13.76 9.60
230 220 210 200 190 180 170 160 150 140 130 120 110 100 90 80 70 60 50 40 30 20 10 0 -10

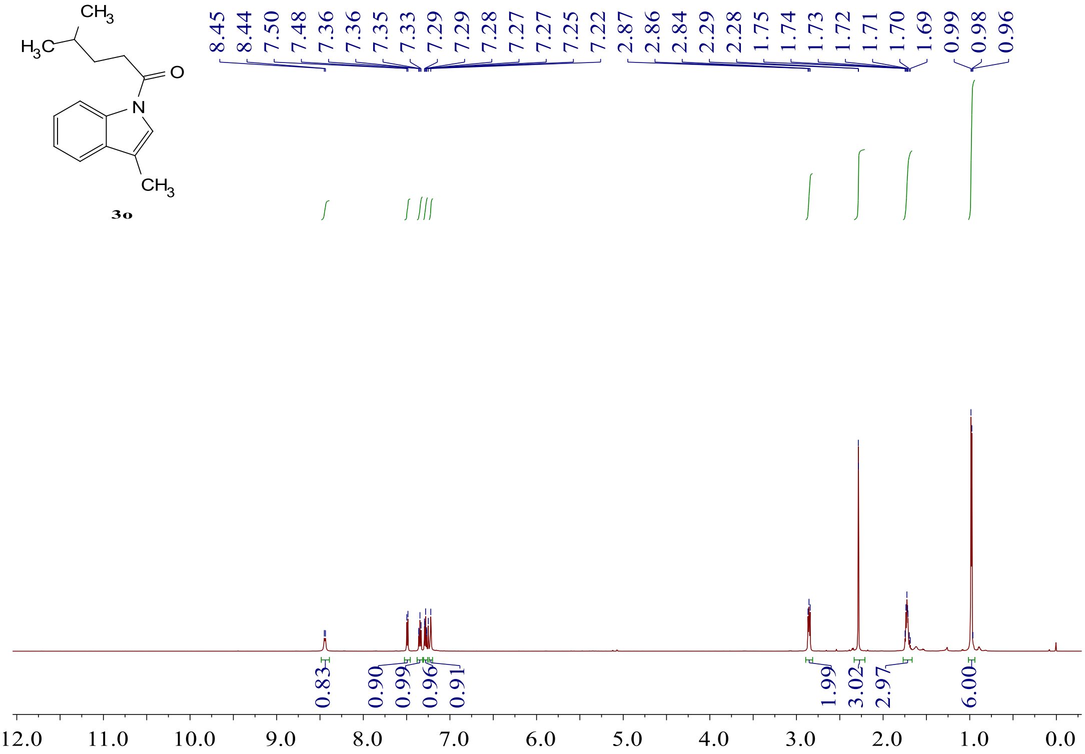
CH3
H3C
O
N
CH3
3o
8.45 8.44 7.50 7.48 7.36 7.36 7.35 7.33 7.29 7.29 7.28 7.27 7.27 7.25 7.22 2.87 2.86 2.84 2.29 2.28 1.75 1.74 1.73 1.72 1.71 1.70 1.69 0.99 0.98 0.96
0.83 0.90 0.99 0.96 0.91
1.99 3.02 2.97 6.00
12.0 11.0 10.0 9.0 8.0 7.0 6.0 5.0 4.0 3.0 2.0 1.0 0.0

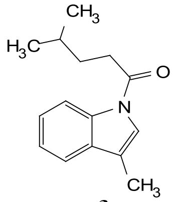

3o

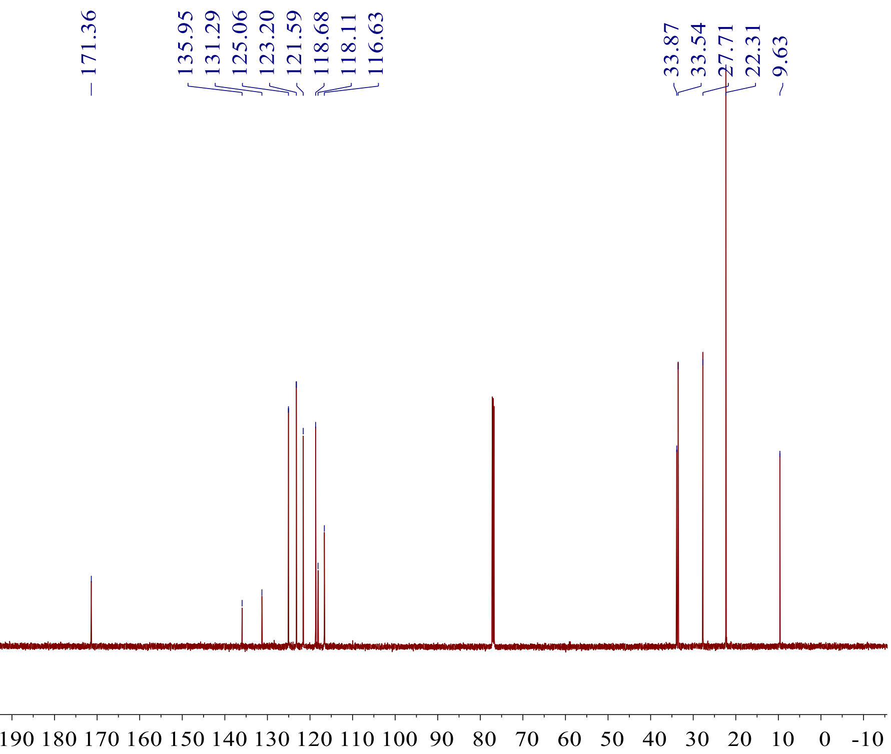
171.36
135.95
131.29
125.06
123.20
121.59
118.68
118.11
116.63
33.87
33.54
27.71
22.31
9.63
230 220 210 200 190 180 170 160 150 140 130 120 110 100 90 80 70 60 50 40 30 20 10 0 -10

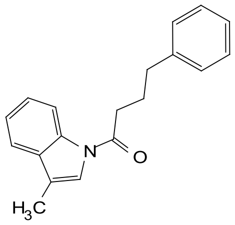

3p

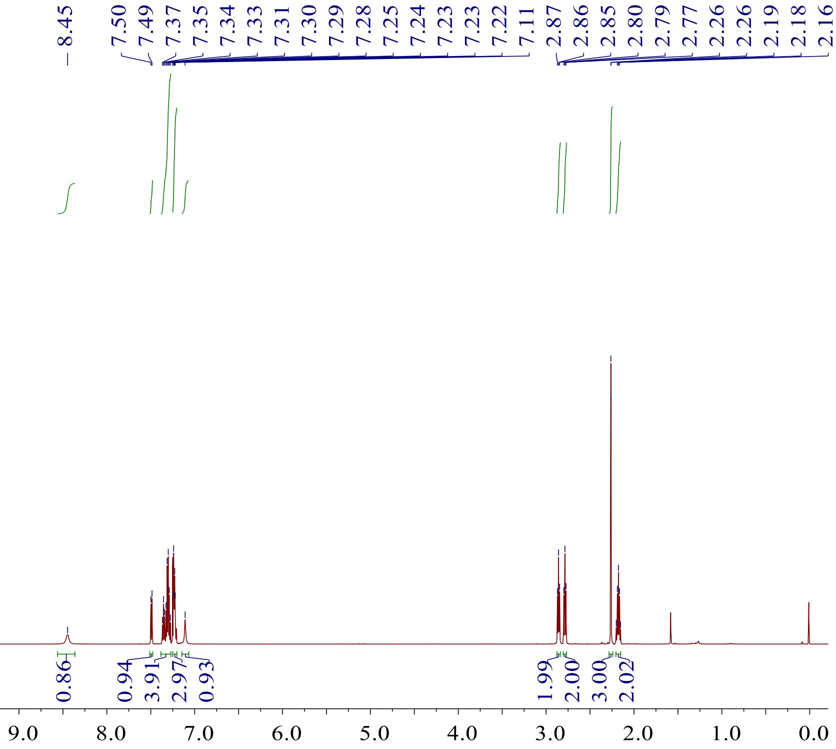
8.45
7.50
7.49
7.37
7.35
7.34
7.33
7.31
7.30
7.29
7.28
7.25
7.24
7.23
7.23
7.22
7.11
2.87
2.86
2.85
2.80
2.79
2.77
2.26
2.26
2.19
2.18
2.16
0.86
0.94
3.91
2.97
0.93
1.99
2.00
3.00
2.02
11.0 10.0 9.0 8.0 7.0 6.0 5.0 4.0 3.0 2.0 1.0 0.0

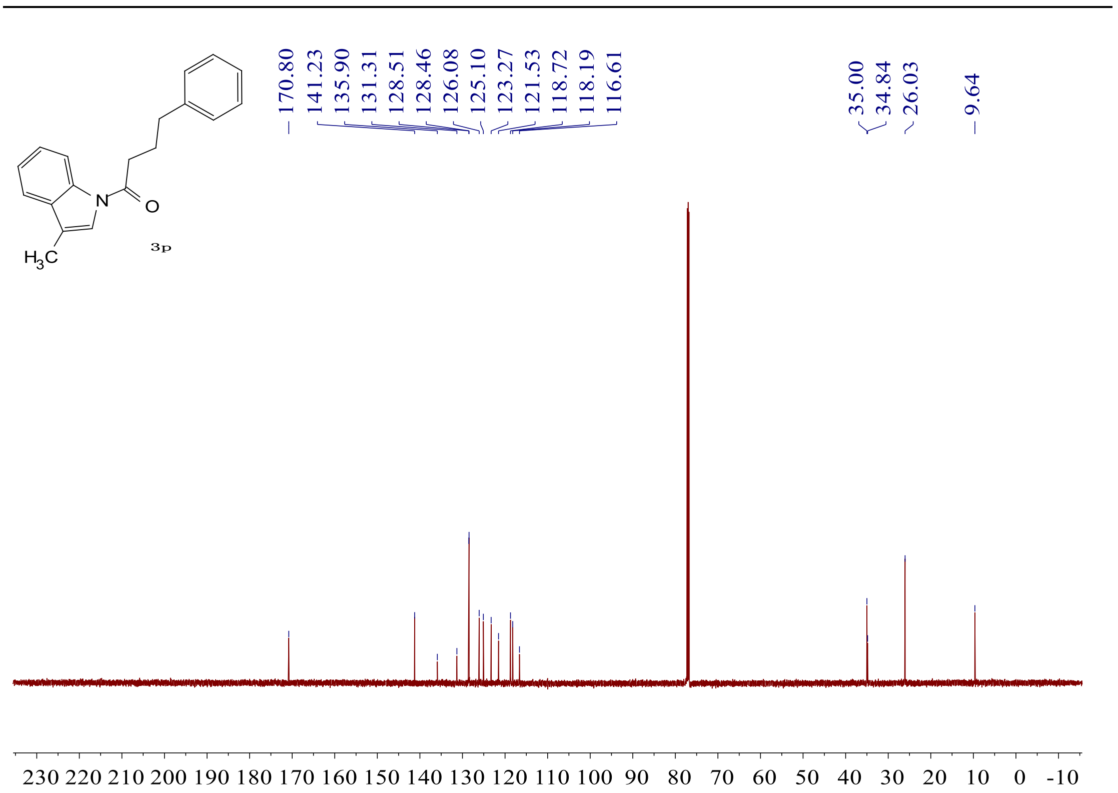
170.80
141.23
135.90
131.31
128.51
128.46
126.08
125.10
123.27
121.53
118.72
118.19
116.61
35.00
34.84
26.03
9.64
O
N
H3C
3p
230 220 210 200 190 180 170 160 150 140 130 120 110 100 90 80 70 60 50 40 30 20 10 0 -10

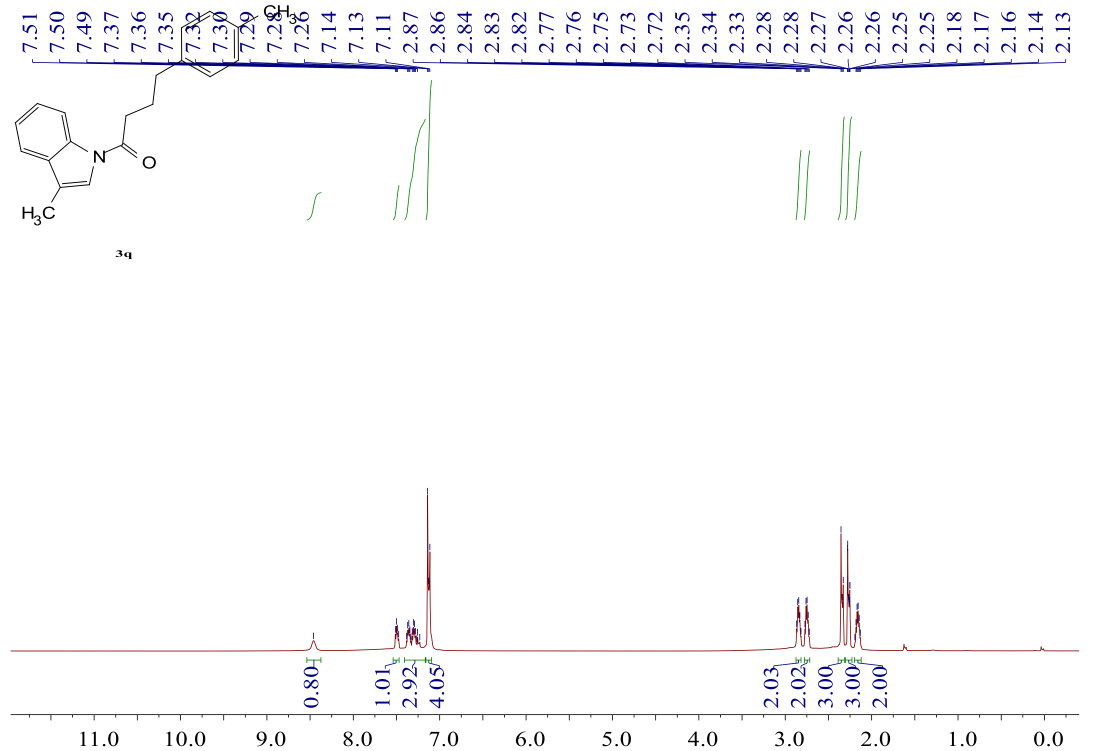
7.51
7.50
7.49
7.37
7.36
7.35
7.32
7.30
7.29
7.27
7.26
7.14
7.13
7.11
2.87
2.86
2.84
2.83
2.82
2.77
2.76
2.75
2.73
2.72
2.35
2.34
2.33
2.28
2.28
2.27
2.26
2.26
2.25
2.25
2.18
2.17
2.16
2.14
2.13
CH3
O
N
H3C
3q
0.80
1.01
2.92
4.05
2.03
2.02
3.00
3.00
2.00
11.0 10.0 9.0 8.0 7.0 6.0 5.0 4.0 3.0 2.0 1.0 0.0

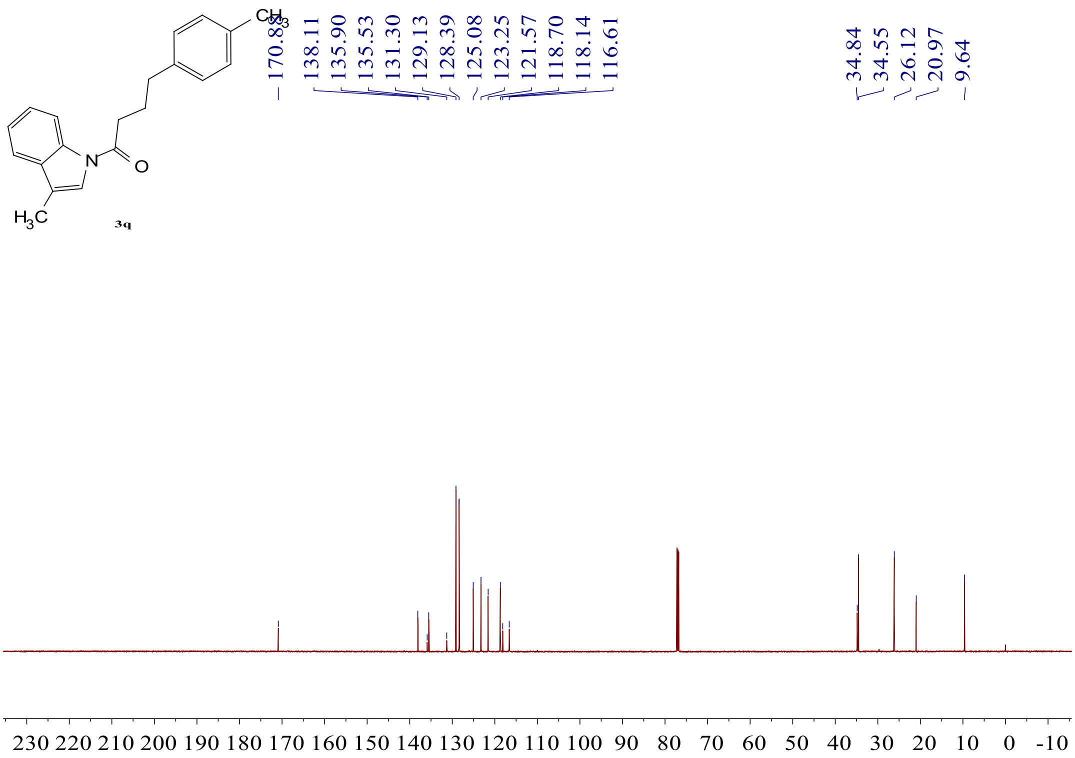
3q
170.83
138.11
135.90
135.53
131.30
129.13
128.39
125.08
123.25
121.57
118.70
118.14
116.61
34.84
34.55
26.12
20.97
9.64
230 220 210 200 190 180 170 160 150 140 130 120 110 100 90 80 70 60 50 40 30 20 10 0 -10

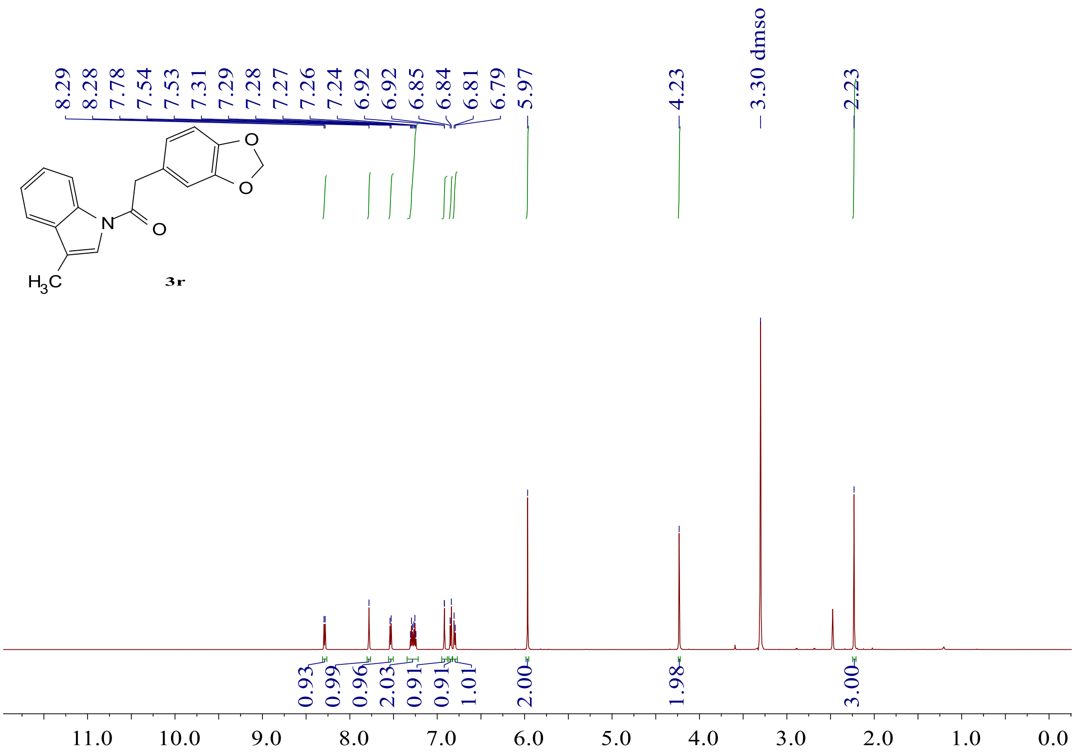
3r
8.29
8.28
7.78
7.54
7.53
7.31
7.29
7.28
7.27
7.26
7.24
6.92
6.92
6.85
6.84
6.81
6.79
5.97
4.23
3.30 dmso
2.23
0.93
0.99
0.96
2.03
0.91
0.91
1.01
2.00
1.98
3.00
11.0 10.0 9.0 8.0 7.0 6.0 5.0 4.0 3.0 2.0 1.0 0.0

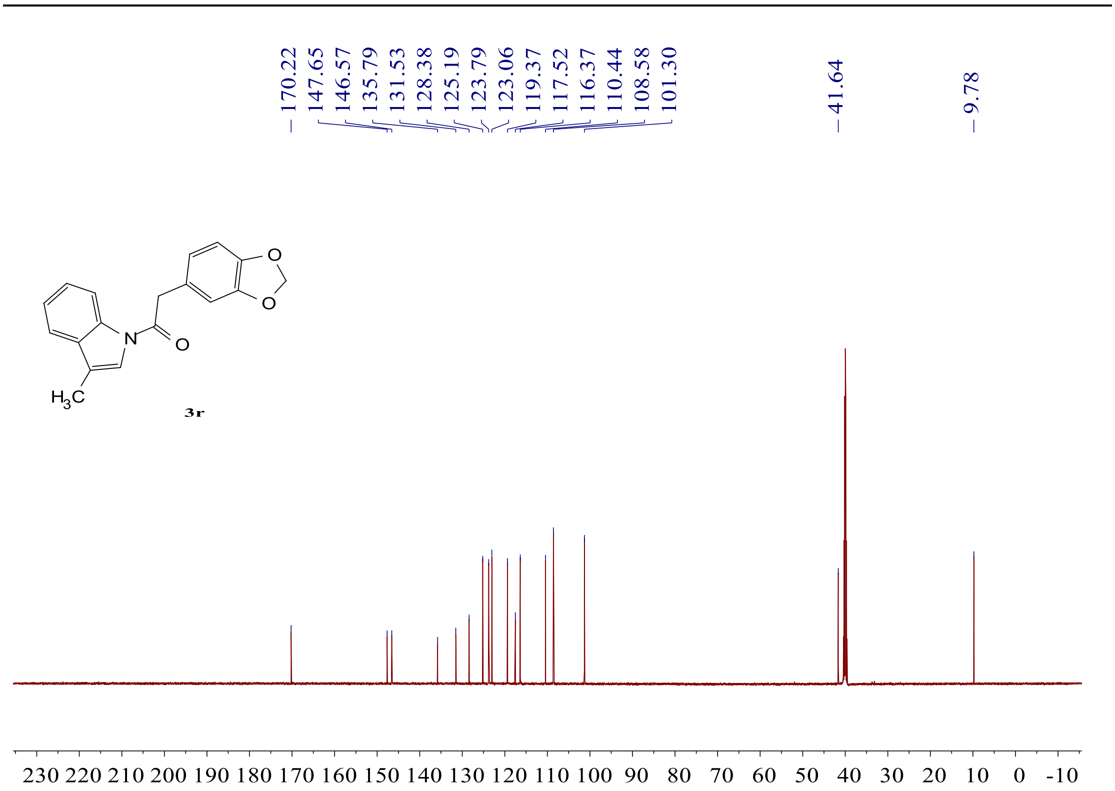
170.22
147.65
146.57
135.79
131.53
128.38
125.19
123.79
123.06
119.37
117.52
116.37
110.44
108.58
101.30
41.64
9.78
O
O
N
O
H_3C
3r
230 220 210 200 190 180 170 160 150 140 130 120 110 100 90 80 70 60 50 40 30 20 10 0 -10

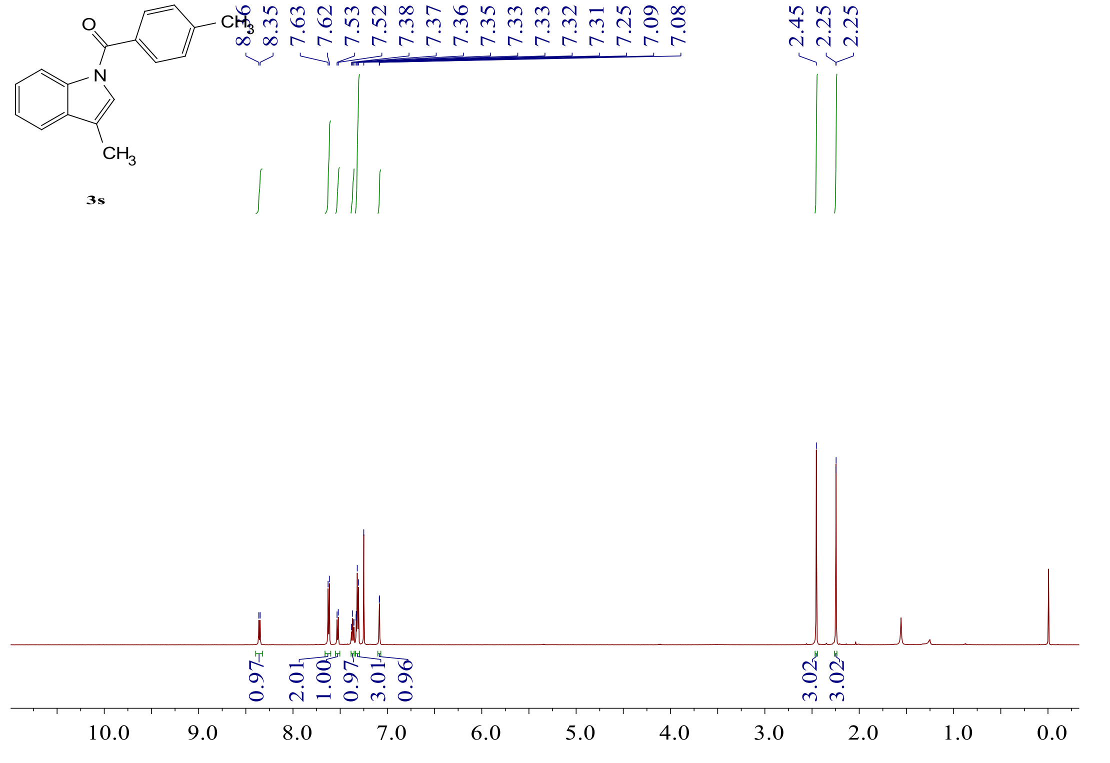
O
CH_3
N
CH_3
3s
8.36
8.35
7.63
7.62
7.53
7.52
7.38
7.37
7.36
7.35
7.33
7.33
7.32
7.31
7.25
7.09
7.08
2.45
2.25
2.25
0.97
2.01
1.00
0.97
3.01
0.96
3.02
3.02
10.0 9.0 8.0 7.0 6.0 5.0 4.0 3.0 2.0 1.0 0.0

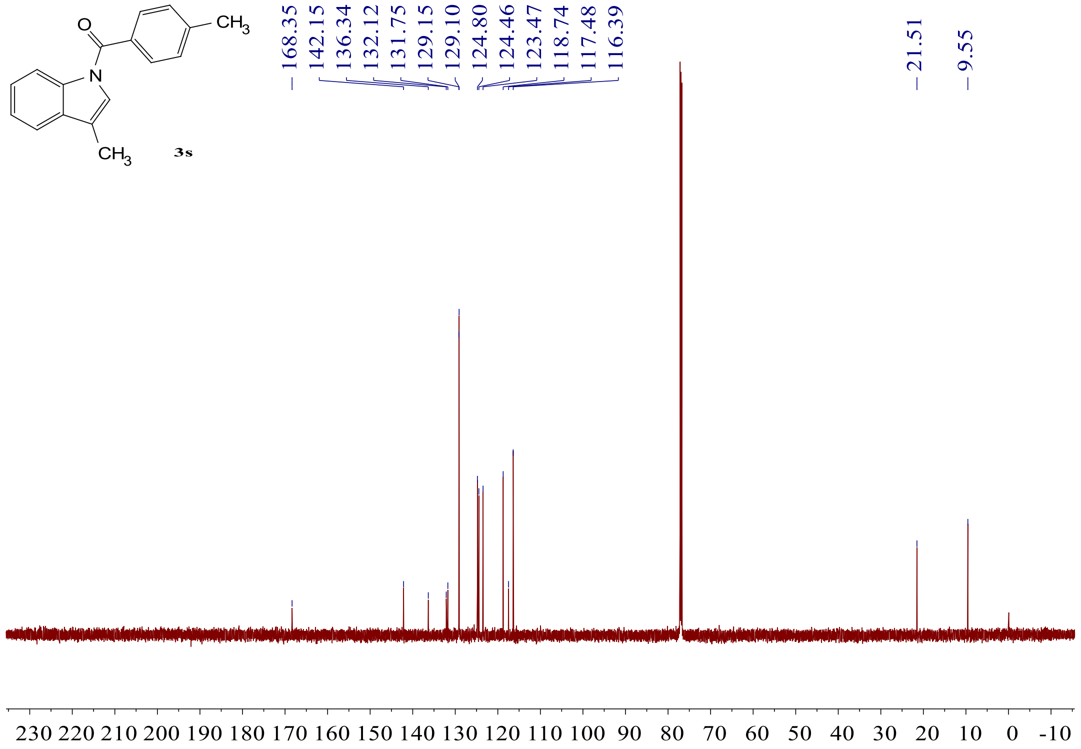
O
N
CH3
168.35
142.15
136.34
132.12
131.75
129.15
129.10
124.80
124.46
123.47
118.74
117.48
116.39
21.51
9.55
230 220 210 200 190 180 170 160 150 140 130 120 110 100 90 80 70 60 50 40 30 20 10 0 -10

3s

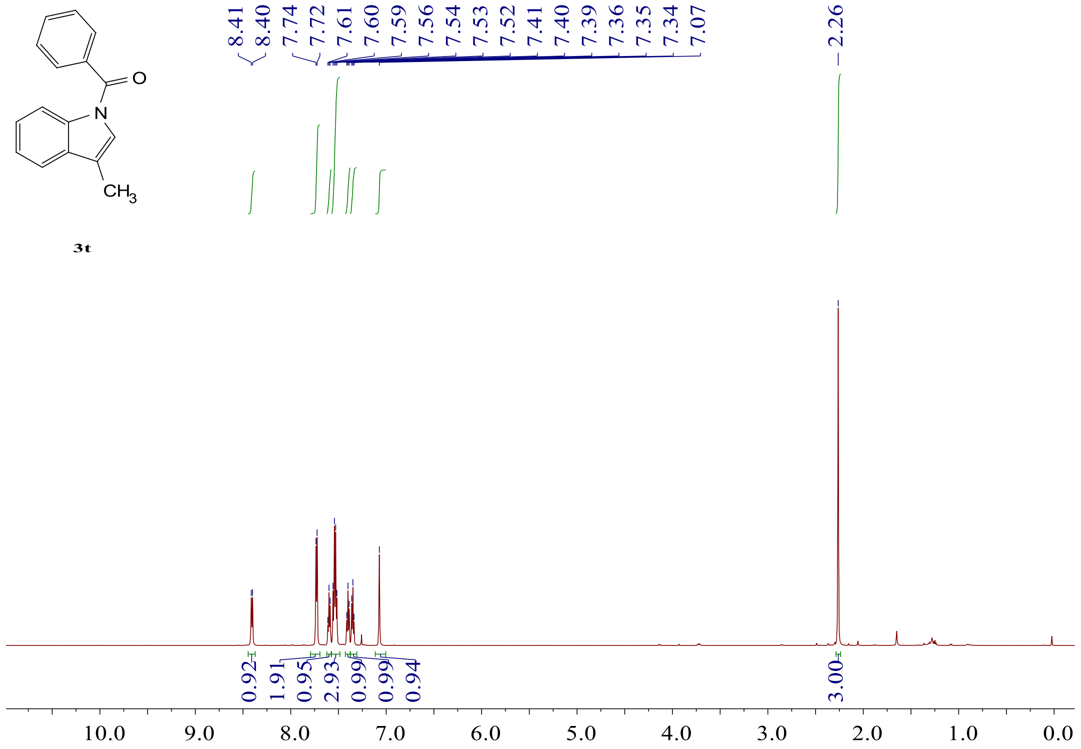
O
N
CH3
8.41
8.40
7.74
7.72
7.61
7.60
7.59
7.56
7.54
7.53
7.52
7.41
7.40
7.39
7.36
7.35
7.34
7.07
2.26
0.92
1.91
0.95
2.93
0.99
0.99
0.94
3.00
10.0 9.0 8.0 7.0 6.0 5.0 4.0 3.0 2.0 1.0 0.0

3t

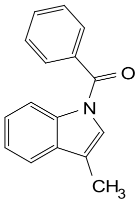
O
N
CH3

3t

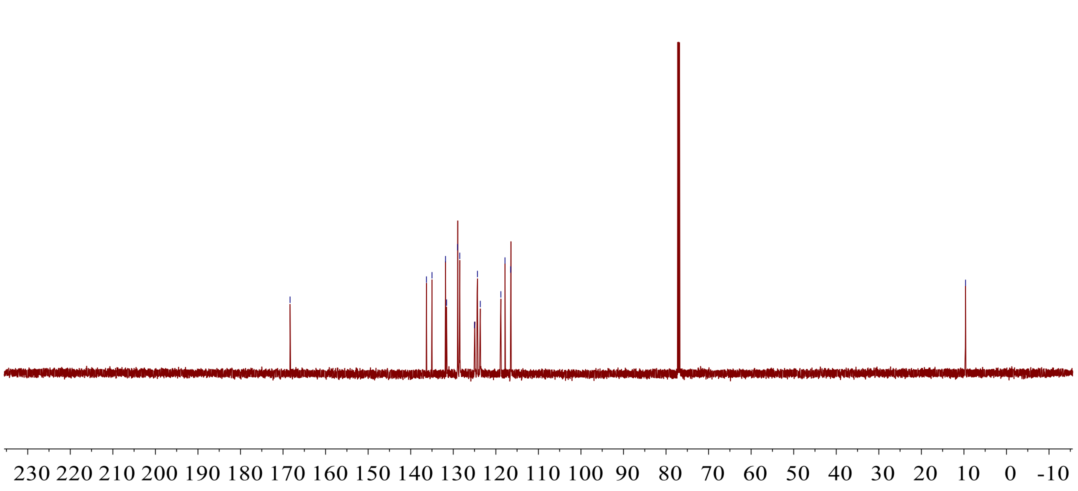
168.34
136.31
135.00
131.82
131.56
128.99
128.48
125.01
124.33
123.65
118.82
117.84
116.50
9.61
230 220 210 200 190 180 170 160 150 140 130 120 110 100 90 80 70 60 50 40 30 20 10 0 -10

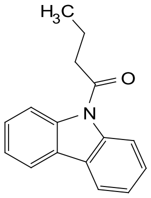
H3C
O
N

3u

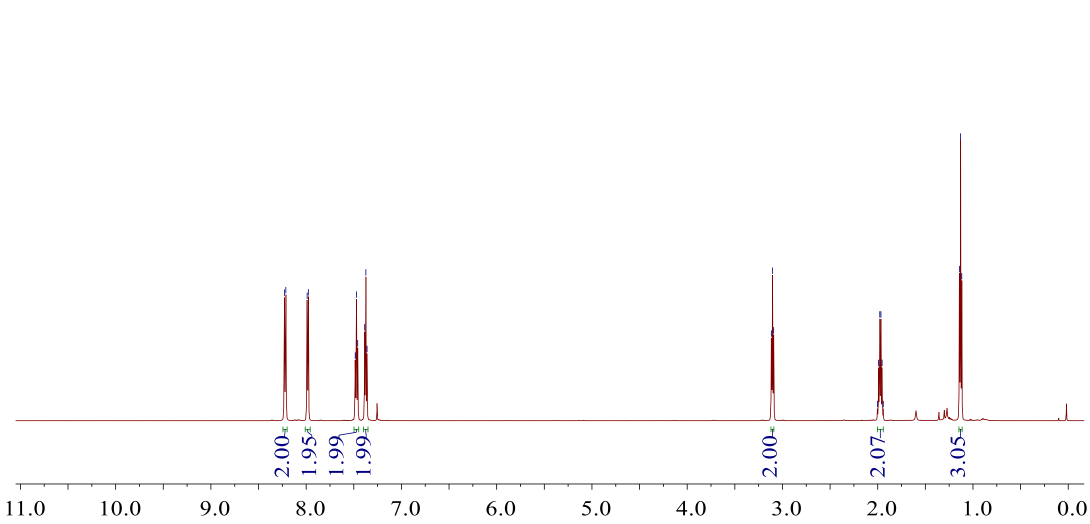
8.23
8.21
7.99
7.98
7.48
7.47
7.46
7.39
7.37
7.36
3.12
3.10
3.09
2.00
1.99
1.98
1.97
1.95
1.94
1.14
1.13
1.12
2.00
1.95
1.99
1.99
2.00
2.07
3.05
11.0 10.0 9.0 8.0 7.0 6.0 5.0 4.0 3.0 2.0 1.0 0.0

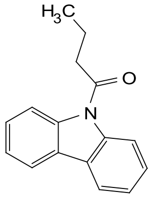

3u

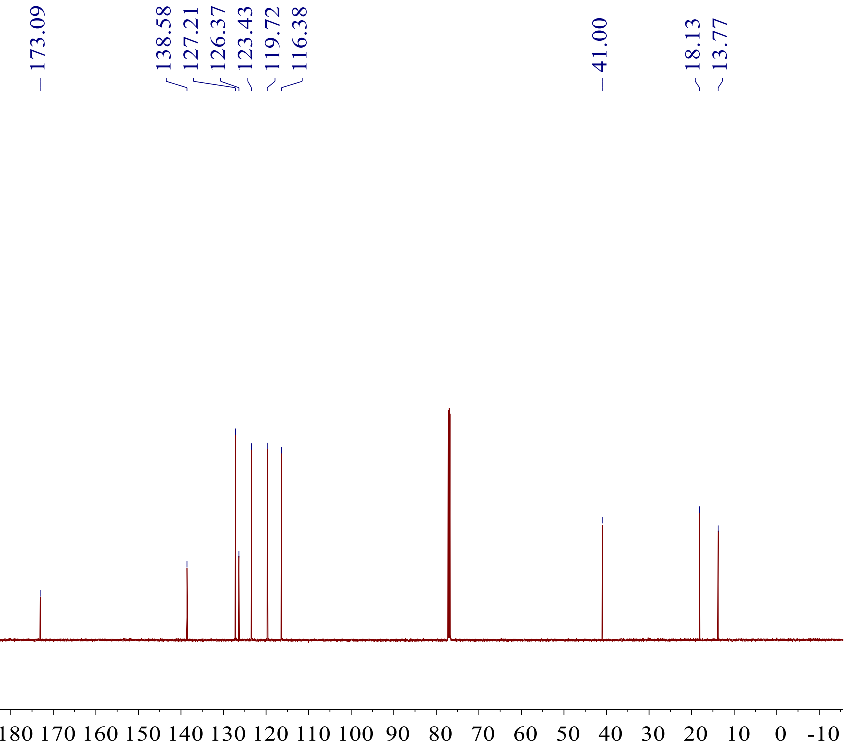

173.09
138.58
127.21
126.37
123.43
119.72
116.38
41.00
18.13
13.77
230 220 210 200 190 180 170 160 150 140 130 120 110 100 90 80 70 60 50 40 30 20 10 0 -10
